# SONDE: a database for exploring the semantics of nouns derived from verbs in French

**DOI:** 10.1007/s11525-026-09463-8

**Published:** 2026-07-07

**Authors:** Richard Huyghe, Justine Salvadori, Rossella Varvara, Lucie Barque, Pauline Haas, Alizée Lombard, Matthieu Monney, Delphine Tribout, Marine Wauquier

**Affiliations:** 1 University of Fribourg, Avenue de l’Europe 20, Fribourg, 1700 Switzerland; 2 University of Turin, Via Verdi 8, Turin, 10124 Italy; 3 University of Pavia, Corso Strada Nuova 65, Pavia, 27100 Italy; 4 Université Sorbonne Paris Nord, 99 avenue Jean-Baptiste Clément, Villetaneuse, 93430 France; 5 LLF CNRS, 8 rue Albert Einstein, Paris, 75013 France; 6 LATTICE CNRS, 1 rue Maurice Arnoux, Montrouge, 92120 France; 7 University of Lille, 42 rue Paul Duez, Lille, 59000 France; 8 STL CNRS, Rue du Barreau, Villeneuve d’Ascq, 59653 France; 9 Paris, France

**Keywords:** Nominalization, Suffix, Polyfunctionality, Lexical aspect, Argument structure, Morphological family, French

## Abstract

Interest in the semantics of word formation has grown significantly in recent years, addressing research questions that require detailed semantic information and extensive data analysis. In this paper, we present the SONDE database, which was developed to investigate the semantics of nouns derived from verbs in French. The database relies on the manual analysis of 5272 deverbal nouns, annotated for their semantic type (taking into account the lexical ambiguity of both bases and derivatives), the lexical aspect of verbs and nouns, and the semantic roles assigned to their arguments. We first describe the construction of the database, including sample selection, the annotation scheme, and an evaluation of annotation reliability. We then analyze the collected data, focusing on (i) the similarity and polyfunctionality of deverbal processes, (ii) the preservation of argument structure and lexical aspect through nominalization, and (iii) the semantic organization of morphological families formed by verbs and their derived nouns. Results show that a network of semantic functions underlies the diversity of deverbal processes, partly shaped by semantically motivated relationships. It appears that the mechanisms of semantic extension that drive polyfunctionality also contribute to the emergence of similarities between processes. Another finding is that the aspectual and argumental properties of base verbs are not necessarily preserved in nominalizations, and that this non-preservation is conditioned by derivational patterns. Finally, the analysis of morphological families reveals distinct structural configurations depending on the semantic alignment of nouns across families. These configurations exhibit varying degrees of defectiveness and are largely determined by the semantic properties of the base verbs.

## Introduction

Interest in the semantics of word formation has increased in recent years (see, e.g., ten Hacken & Thomas, 2013; Rainer et al., 2014; Bauer et al., 2015; Kotowski & Plag, 2023). Common topics of investigation include the polysemy of derivational processes (Melloni, 2011; Schulte, 2015a; Plag et al., 2018; a.o.), semantic differences between competing processes (Naccarato, 2019; Huyghe & Wauquier, 2021; Nagano, 2023; a.o.), the semantic transparency of complex words (Schäfer, 2018; Günther & Marelli, 2019; Libben et al., 2020; a.o.), the transfer of cross-categorial semantic properties between bases and derivatives (Fábregas et al., 2012; Ignjatović, 2016; Lieber, 2023; a.o.), and the semantic organization of derivational paradigms (Fradin, 2020; Laks, 2024; Sanacore et al., 2024; a.o.). Providing reliable answers to research questions on the semantic aspects of word formation requires detailed semantic information about large amounts of data, which can be challenging to obtain. On the one hand, manually curated databases are often limited in the number of complex words or processes they cover, restricting the scope for broad comparisons or quantitative generalization. On the other hand, semantic information automatically obtained on a large scale through computational methods is often too coarse or imprecise to address key research questions. While distributional semantics tools have been successfully used in derivational morphology for their capacity to handle extensive datasets (Marelli & Baroni, 2015; Padó et al., 2016; Stupak & Baayen, 2022; Bonami & Guzmán Naranjo, 2023; Schäfer, 2025; a.o.), they assess word meaning based on distributional similarity, without directly describing their semantic content. In their current state of development, these methods provide limited insight into fine-grained features related to polysemy, semantic roles, lexical aspect or scalar structure, for instance.

In this study, we examine the case of verb-to-noun derivation, an area that concentrates many of the debates about the semantics of derivational processes, yet suffers from the limitations described above. We focus on nouns derived from verbs in French, which have been extensively studied in relation to specific processes but still lack a unified description and comprehensive analysis. Existing morphological resources, such as Nomage (Balvet et al., 2011), VerNom (Missud et al., 2020) and Démonette-2.0 (Namer et al., 2023), either do not include detailed semantic information about deverbal nouns, or only focus on a limited number of nominalizing processes. To provide an overview of the semantic properties of deverbal nouns in French, we developed a database named SONDE,^1^ which relies on the manual analysis of a sample of 5272 French deverbal nouns, categorized according to their semantic type, the lexical aspect of both verbs and nouns, and the semantic roles assigned to their arguments. The database was designed to meet three primary objectives. The first is formal exhaustiveness, aiming to cover virtually all processes used to form deverbal nouns in French, regardless of their productivity. The second is to provide a nuanced semantic description of both derived nouns and base verbs, distinguishing between the various meanings of each derivative and linking them to specific verb meanings. The third objective is to document complete morphological families of deverbal nouns—i.e., all nouns derived from each verb—allowing for further exploration of morphological paradigms in verb-to-noun derivation.

This paper is divided into two main sections. In Sect. 2, we describe the creation of the database and discuss methodological decisions related to sample selection, principles of semantic description, and the annotation procedure. In Sect. 3, we analyze the collected data, with a focus on (i) the similarity and polyfunctionality of deverbal processes, (ii) the preservation of argument structure and lexical aspect in nominalizations, and (iii) the semantic organization of morphological families formed by verbs and deverbal nouns.

## Creation of the database

The SONDE database was developed by a team of 9 researchers in the context of a research project on the semantics of nouns derived from verbs. In this section, we present the method used to create the database, beginning with the sampling of French deverbal nouns (2.1). We then detail the principles of semantic analysis applied to both base verbs and derived nouns (2.2), and finally describe the annotation procedure (2.3).

### Sampling

The creation of SONDE was primarily based on the identification of 42 suffixes and 4 types of conversion involved in the derivation of nouns from verbs in French. The list of suffixes was compiled from prior research on specific processes (see, e.g., Kelling, 2001 for -*age* and -*ment*; Schnedecker & Aleksandrova, 2018 for -*aire*; Burdy, 2013 for *-aison*; Knittel, 2016 for -*ance*; Dal, 1999 for -*et* and -*ette*; Namer & Villoing, 2008 for -*oir*), as well as studies aiming at a complete description of derivation in French (Dubois, 1962; Thiele, 1987; Apothéloz, 2002), and extensive derivational resources such as Démonette-2.0 (Namer et al., 2023). While orthographic and allomorphic variants (e.g., -*ier* and -*er*, -*ance* and -*ence*) were grouped together, suffixes differing by the gender of the derivative (e.g., *-ard* and *-arde*, *-et* and *-ette*) were analyzed separately, considering that gender specification is a feature of nominalization processes. Following this rationale, we treated the masculine and feminine versions of the same suffixal forms as distinct affixes, independently of their potential semantic equivalence. For example, we distinguished two suffixes for both -*eur* and -*aire* depending on whether they form masculine and feminine nouns, regardless of their ability to produce epicene nouns. To ensure a comprehensive description of deverbal processes, we included verb-noun conversions alongside suffixed forms. Specifically, 4 types of conversion were considered, distinguished by their formal features and the verb stem on which they are based: conversion based on the verb stem “0”, which roughly corresponds to the present singular form (Tribout, 2012); conversion based on the verb stem “12”, which underlies past participle formation (Bonami & Boyé, 2003); conversion based on the verb stem “13”, historically derived from Latin supine and only used in suffixation (Kerleroux, 2007; Bonami et al., 2009); and substantivized infinitives, despite debates regarding their status as true conversions (see Kerleroux, 1996). Table 1 presents the complete list of these morphological processes with examples.

**Table 1:** Morphological processes described in SONDE

	Noun	Verb
Suffixation		
-*ade*	*ruade* ‘kicking’	*ruer* ‘kick’
-*age*	*atterrissage* ‘landing’	*atterrir* ‘land’
-*ail*	*épouvantail* ‘scarecrow’	*épouvanter* ‘scare’
-*aille*	*sonnaille* ‘bell’	*sonner* ‘ring’
-*ain*	*écrivain* ‘writer’	*écrire* ‘write’
-*aire* (feminine)	*locataire* ‘tenant’	*louer* ‘rent’
-*aire* (masculine)	*commentaire* ‘comment’	*commenter* ‘comment’
-*aison*	*livraison* ‘delivery’	*livrer* ‘deliver’
-*ance*/-*ence*	*provenance* ‘origin’	*provenir* ‘come from’
-*ant*/-*ent*	*militant* ‘militant’	*militer* ‘campaign’
-*ante*/-*ente*	*dirigeante* ‘director’	*diriger* ‘manage’
-*ard*	*pillard* ‘pillager’	*piller* ‘pillage’
-*arde*	*scribouillarde* ‘penpusher’	*scribouiller* ‘scribble’
-*asse*	*liasse* ‘bundle’	*lier* ‘tie’
-*eau*/-*ereau*	*traîneau* ‘sled’	*traîner* ‘drag’
-*elle*/-*erelle*	*sauterelle* ‘grasshopper’	*sauter* ‘jump’
-*er*	*surfer* ‘surfer’	*surfer* ‘surf’
-*er*/-*ier*	*héritier* ‘heir’	*hériter* ‘inherit’
-*ère*/-*ière*	*conseillère* ‘adviser’	*conseiller* ‘advise’
-*eresse*	*chasseresse* ‘huntress’	*chasser* ‘hunt’
-*erie*	*cajolerie* ‘cuddle’	*cajoler* ‘cuddle’
-*et*	*sifflet* ‘whistle’	*siffler* ‘whistle’
-*ette*	*causette* ‘chat’	*causer* ‘chat’
-*eur* (feminine)	*valeur* ‘value’	*valoir* ‘be worth’
-*eur* (masculine)	*assureur* ‘insurer’	*assurer* ‘insure’
-*eure*	*gouverneure* ‘governor’	*gouverner* ‘govern’
-*euse*	*photocopieuse* ‘photocopier’	*photocopier* ‘photocopy’
-*in*	*pétrin* ‘kneading machine’	*pétrir* ‘knead’
-*ine*	*tétine* ‘teat’	*téter* ‘suckle’
-*ing*	*briefing* ‘briefing’	*briefer* ‘brief’
-*ion*	*indication* ‘indication’	*indiquer* ‘indicate’
-*is*	*dégueulis* ‘vomit’	*dégueuler* ‘throw up’
-*ise*	*hantise* ‘terror’	*hanter* ‘haunt’
-*ment*	*chuchotement* ‘whispering’	*chuchoter* ‘whisper’
-*oir*/-*oire* (masculine)	*mouroir* ‘place to die’	*mourir* ‘die’
-*oire* (feminine)	*écumoire* ‘skimmer’	*écumer* ‘skim’
-*on*	*juron* ‘swear word’	*jurer* ‘swear’
-*onne*	*forgeronne* ‘blacksmith’	*forger* ‘forge’
-*ot*	*binot* ‘hoe’	*biner* ‘hoe’
-*ote*/-*otte*	*jugeote* ‘common sense’	*juger* ‘judge’
-*rice*	*sculptrice* ‘sculptor’	*sculpter* ‘sculpt’
-*ure*	*craquelure* ‘crack’	*craqueler* ‘crack’
Conversion		
Stem 0	*ébauche* ‘preliminary draft’	*ébaucher* ‘start’
Stem 12	*fumée* ‘smoke’	*fumer* ‘smoke’
Stem 13	*postulat* ‘postulate’	*postuler* ‘postulate’
Infinitive	*sourire* ‘smile’	*sourire* ‘smile’

Our initial goal was to compile a sample of at least 4000 verb-noun pairs, in which the verbs and nouns are formally and semantically related in synchrony. The sample was assembled from FRCOW16A (Schäfer & Bildhauer, 2012; Schäfer, 2015), a large web-based corpus of contemporary French containing 10.8 billion tokens. This corpus includes nouns listed in standard dictionaries (e.g., *brûlure* ‘burn’, *glissade* ‘slip’, *tricherie* ‘cheating’) as well as neologisms and nonce words (e.g., *noctambulation* ‘nocturnal wandering’, *pendouillement* ‘dangling’, *roucoulage* ‘cooing’). Nouns formally related to lemmatized verbs in the corpus were automatically extracted based on concatenation with suffixes and conversion exponents. Regular expressions were formulated for the 46 previously identified processes, taking into account both standard verbal stems and 65 possible allomorphic patterns, such as those in (1).

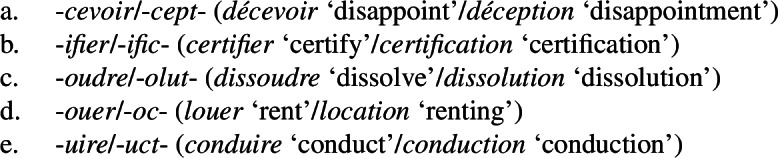

The automatic extraction process generated 59,353 candidate verb-noun pairs, including a substantial amount of noise. A controlled manual selection was then conducted to identify relevant pairs. The basic condition for selection was that a semantic relationship could be established in the current state of the language between at least one meaning of the noun and one meaning of the related verb. For instance, the pair *visionner* ‘view’/*visionneuse* ‘viewer’ was retained, as the noun *visionneuse* denotes an instrument used for viewing something. Conversely, the pair *peigner* ‘comb’/*peignoir* ‘robe’ was excluded because *peignoir* no longer maintains a semantic link to its base verb, despite historical connections.

Nouns with a potentially multimotivated morphological structure were filtered according to their semantic analyzability as deverbal nouns—whether they were prefixed-suffixed nouns with ambiguous construction pathways or nouns possibly suffixed from either a verbal or a nonverbal base. In such cases, an additional criterion was applied to confirm their potential deverbal status: the nouns had to follow a derivational pattern of the type *V* → *N-x* supported by at least two analyzable examples of *N-x* that are verb-derived, monosemous, and of the same semantic type. For example, instrumental nouns ending in -*ette*, such as *moulinette* ‘food mill’, were included in the database despite the theoretical possibility of a nominal base (*moulin* ‘mill’ + -*ette* → *moulinette*). This inclusion was justified by the existence of monosemous deverbal nouns of the same semantic category (e.g., *allumette* ‘match’, *chaufferette* ‘heater’) with unequivocally verbal bases. Derivational patterns were identified using both the literature on suffixation and lexicographic sources (e.g., *Le Robert méthodique*, Rey-Debove, 1985; *Le Petit dictionnaire des suffixes du français*, Editions Le Robert, n.d.-b). As far as conversions are concerned, substantivized infinitives (e.g., *déjeuner*_V_ ‘have lunch’/*déjeuner*_N_ ‘lunch’) were systematically retained provided that they were semantically related to the verbs. Conversions from stem 0 were selected only if they denoted an eventuality in at least one sense (e.g., *danser* ‘dance’/*danse* ‘dance’), and excluded if they referred to entities (e.g., *poivrer* ‘put pepper in sth’/*poivre* ‘pepper’). Finally, it was decided to restrict the database to the standard lexicon. Nouns or nominal senses classified as regionalisms, archaisms, or technical terms were not considered, as their semantic analysis is often hindered by limited intuitive understanding. Identifying a noun as a regionalism, an archaism or a technical term was based on the information provided by two established dictionaries, *Le Petit Robert* (Editions Le Robert, n.d.-a) and *Le Trésor de la Langue Française informatisé* (ATILF - CNRS & Université de Lorraine, n.d.) for standard entries, and web resources for neologisms.

Following these conditions, the manual selection of deverbal nouns was conducted by 6 team members in 3 distinct steps. In the first step, raw lists generated by the automatic extraction were exhaustively filtered for 36 relatively underrepresented morphological processes in FRCOW16A—specifically, those with less than 950 candidate pairs (e.g., -*ade*, -*ail*, -*ard*, -*is*, -*ette*). In the second step, morphological families were completed by adding nouns created through the 10 remaining processes, namely suffixations in -*age*, -*ance*, -*ant*, -*ante*, -*eur* (masculine), -*ion*, -*ment*, -*ure*, and conversions from stems 0 and 12. For example if *guillotinade* ‘guillotining’ was selected in the first phase, then *guillotinage* ‘guillotining’, *guillotinement* ‘guillotining’ and *guillotineur* ‘guillotiner’ were subsequently added to the dataset. In the third step, 2000 random verb-noun pairs involving the 10 processes with 950 or more candidates were added to enhance their representation in the database, and complete morphological families were also formed at this stage.^2^ The final sample includes 5272 nouns and 1709 verbs. Note that our sampling procedure was intended to ensure sufficient representation of weakly productive processes for accurate description and quantitative analysis. However, this approach also causes these processes to be overrepresented in the sample compared to highly productive processes.

### Semantic description

For each verb-noun pair included in the sample, we annotated a series of features related to (i) the semantic type of the noun, (ii) the lexical aspect of both the verb and the noun, (iii) the semantic roles assigned by the verb and the noun to their arguments. Multiple entries were created for ambiguous nouns and paired with specific meanings of the base verbs. The description of the verb-noun pairs was based on explicit definitions of semantic features and on linguistic tests, which are detailed in the annotation guidelines provided in the supplementary materials of the paper. In this subsection, we outline the general principles of semantic analysis applied in the annotation process.

#### Semantic types

The analysis of the 5272 deverbal nouns in the database relies on their classification into distinct semantic categories. Existing classification models for deverbal nouns (see, e.g., Ježek, 2007; Fradin, 2012; Schulte, 2015b; Lieber, 2016; Kawaletz, 2023) often conflate two important dimensions of meaning: the ontological nature of the referent (e.g., event, state, animate) and the semantic relation with the eventuality described by the base verb (e.g., patient, result, instrument). These two components—ontological and relational—are distinct in nature and compatible rather than mutually exclusive (Huyghe, 2015). A single ontological type can be associated with different relational types, and vice versa. For instance, nominalizations denoting artefacts can instantiate different relations with the base verb, denoting a result, an instrument, or a location, as illustrated in (2). Conversely, nouns that describe the result of an eventuality may refer to a variety of entities or eventualities, including artefacts, psychological states, or animate entities, as shown in (3).

(2)

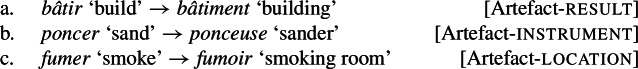

(3)

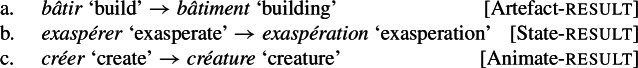

Based on the distinction between ontological and relational types, we described each deverbal noun using a combination of two semantic types.^3^ We applied an ontological classification that is relevant to all kinds of nouns, whether morphologically simple or complex, to provide an interoperable description that makes deverbal nouns comparable with the rest of the nominal lexicon. 15 ontological simple types were distinguished based on specific distributional properties identified in the literature (Godard & Jayez, 1996; Flaux & Van de Velde, 2000; Haas et al., 2023; a.o.). For example, a derivative was classified as Event if it could be used as the subject of *se produire* ‘occur’ or *avoir lieu* ‘take place’, or as the object of *procéder* ‘proceed’, *effectuer* ‘perform’ or *accomplir* ‘accomplish’. Alternatively, it was classified as Artefact if it could serve as the object of *fabriquer* ‘make’, *déchirer* ‘tear’, *construire* ‘build’ or *confectionner* ‘make’, followed by a material modifier (e.g., *en bois* ‘made of wood’). The distributional tests were applied to individual nominal senses, according to the decision tree proposed by Haas et al. (2023). Nouns whose ontological type resists clear attribution due to ontologically unspecified denotation, such as *composant* ‘component’, *mélange* ‘mix’ and *variante* ‘variant’, received the label “NA”.

In addition to the 15 simple types, the classification also includes 7 complex types, each formed by a combination of two simple ontological types. These complex types account for nouns with a hybrid semantic structure (see Pustejovsky, 1995; Cruse, 1995; Asher, 2011; a.o.). Unlike standard polysemes, complex-type nouns support type copredication. They are characterized by two simple ontological types but also compatible within the same context with predicates distinctive of each type. For example, the noun *déclaration* ‘statement’ in (4) instantiates a complex Cognitive*Event type. The eventive facet is selected by *effectuer* ‘perform’ and the cognitive facet by *selon lequel P* ‘according to which P’.

(4)

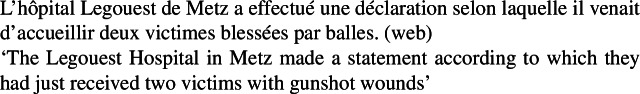

An overview of the 15 simple types and 7 complex types is provided in Table 2. We additionally took into account that the nouns could be collective, denoting plural entities in the singular form, and introduced the option of refining any of the ontological types with a Collective label (abbreviated as ⋅Coll). Examples of collective deverbal nouns include nouns such as *assistance* ‘assistance’, *parure* ‘jewelry’ and *tuerie* ‘massacre’, which denote groups of entities or eventualities, and were analyzed as Animate⋅Collective, Artefact⋅Collective and Event⋅Collective, respectively.

**Table 2:** Ontological types

	Abbreviation	Example
Simple ontological type		
Animate	Anm	*collaboratrice* ‘colleague’
Artefact	Art	*bouilloire* ‘kettle’
Cognitive	Cog	*corrélat* ‘correlate’
Disease	Dis	*couvade* ‘sweating sickness’
Domain	Dom	*jardinage* ‘gardening’
Event	Evt	*inspection* ‘inspection’
Financial	Fin	*redevance* ‘license-fee’
Institution	Ist	*association* ‘society’
Natural	Nat	*mâchoire* ‘jawbone’
Phenomenon	Phn	*senteur* ‘scent’
Property	Ppt	*persévérance* ‘perseverance’
Quantity	Qua	*lichette* ‘lick’
State	Sta	*agacement* ‘irritation’
Time	Tim	*couchant* ‘setting sun’
NA	NA	*échappatoire* ‘way out’
Complex ontological type		
Artefact*Cognitive	Art*Cog	*circulaire* ‘memorandum’
Artefact*Institution	Art*Ist	*restaurant* ‘restaurant’
Cognitive*Event	Cog*Evt	*témoignage* ‘testimony’
Event*Financial	Evt*Fin	*paiement* ‘payment’
Event*Natural	Evt*Nat	*inflammation* ‘inflammation’
Event*Phenomenon	Evt*Phn	*crissement* ‘squeaking’
Event*State	Evt*Sta	*disparition* ‘disappearance’

The relational classification includes 18 semantic types that represent potential participants in eventualities. These types were adapted from existing frameworks on semantic roles, particularly VerbNet (Kipper-Schuler, 2005) and LIRICS (Petukhova & Bunt, 2019). Each semantic role was explicitly defined and the assignment of relational types was based on these definitions. For instance, the noun *parieur* ‘bettor’ was assigned the relational type agent because it refers to an individual who intentionally performs the action denoted by the verb *parier* ‘bet’. Similarly, the noun *hachoir* ‘mincer’ was categorized as instrument, as it refers to an entity used to perform the action expressed by the verb *hacher* ‘mince’. The full list of relational types and their definitions is provided in Table 3. Alongside major semantic roles such as agent, instrument, and theme, the classification also includes a transposition type, designed to account for derivatives that express a similar type of eventuality as their base verb (Beard, 1995; Spencer, 2013; ten Hacken, 2015; Lieber, 2015; a.o.). For instance, *licenciement* ‘dismissal’ and *méfiance* ‘distrust’ were analyzed as transpositional derivatives because they closely parallel the eventualities denoted by their base verbs *licencier* ‘dismiss’ and *se méfier* ‘distrust’. Transposition is considered here as a broad category, focusing mainly on the preservation of dynamicity between the base verb and the derived noun, regardless of potential shifts in other aspectual features, such as durativity or telicity. A dynamic or stative noun need only be derived from a dynamic or stative verb, respectively, to be analyzed as transpositional.

**Table 3:** Relational types

Relational type	Abbreviation	Definition	Verb	Noun
agent	agt	Entity that brings about an event intentionally	*forger* ‘forge’	*forgeron* ‘blacksmith’
beneficiary	ben	Entity that receives or is dispossessed of something, or that is advantaged or disadvantaged by an eventuality	*hériter* ‘inherit’	*héritier* ‘heir’
cause	cau	Entity that initiates an eventuality (not necessarily intentionally), or is the reason why an eventuality occurs	*catalyser* ‘catalyze’	*catalyseur* ‘catalyst’
destination	des	Endpoint in a change of location	*cracher* ‘spit’	*crachoir* ‘spittoon’
experiencer	exp	Entity that is in or enters a particular state in relation to a psychological, perceptive or physiological stimulation	*paniquer* ‘panic’	*paniquard* ‘panicker’
extent	ext	Extensive value related to an event, or measurable magnitude of a change of state or location	*contenir* ‘contain’	*contenance* ‘capacity’
instrument	ins	Entity that is manipulated in order to perform an action	*percer* ‘drill’	*perceuse* ‘drill’
location	loc	Entity that serves as a landmark to locate another entity or an event	*patiner* ‘skate’	*patinoire* ‘skating rink’
manner	man	The way an action is performed, or the intensity of a state	*prononcer* ‘pronounce’	*prononciation* ‘pronunciation’
path	pth	Trajectory followed during a change of location	*dévier* ‘deviate’	*déviation* ‘deviation’
patient	pat	Entity that undergoes a change of structure or condition	*mourir* ‘die’	*mourant* ‘dying person’
pivot	pvt	Entity that is attributed a property, or is in a non-stimulated condition	*composer* ‘compose’	*composant* ‘component’
result	res	Entity that is created through an event	*égratigner* ‘scratch’	*égratignure* ‘scratch mark’
source	src	Starting point in a change of location	*plonger* ‘dive’	*plongeoir* ‘diving board’
stimulus	sti	Entity that causes a psychological, perceptive or physiological state	*épouvanter* ‘frighten’	*épouvantail* ‘scarecrow’
theme	thm	Entity that is in a certain location or changes location	*traîner* ‘drag’	*traîneau* ‘sled’
topic	tpc	Entity that is a subject of thought, discussion or cognitive activity	*supposer* ‘suppose’	*supposition* ‘supposition’
transposition	tsp	Eventuality denoted by the base lexeme	*atterrir* ‘land’	*atterrissage* ‘landing’

Note that nouns with figurative extensions that are no longer related to the base verb could not be assigned a standard relational type. Such cases were annotated with a composite label that includes (i) the relational type of the source sense and (ii) a figurative tag to indicate their detachment from the base verb. For example, the noun *pilon* derived from *piler* ‘crush’, which is polysemous between an instrumental meaning (‘pestle’) and a metaphorical meaning (‘chicken leg’), was assigned two entries in the database. The first entry *pilon*_1_ was annotated as Artefact-instrument, while the second entry *pilon*_2_ was annotated as Artefact-(instrument⋅)figurative.

#### Lexical aspect

Lexical aspect was examined for verbs and eventuality-denoting nouns (while not applying to entity-denoting nouns). It was decomposed into four basic features—dynamicity, durativity, telicity, and post-phase—and analyzed using linguistic tests proposed in the literature (Vendler, 1967; Dowty, 1979; Rothstein, 2004; Haas et al., 2008; Heinold, 2010; Meinschaefer, 2016; a.o.). For instance, durativity for verbs was determined based on their compatibility with *commencer* ‘begin’, *continuer* ‘continue’ or *arrêter* ‘stop’, or with durative adverbials introduced by *en* ‘in’ or *pendant* ‘for’. Durativity for nouns was assessed by testing whether they could serve as the subject of *durer* ‘last’ or *se dérouler* ‘unfold’, or combine with durative modifiers (e.g., *une réunion de deux heures* ‘a two-hour meeting’).

Telicity, which is known to be coerced by non-delimited internal arguments (e.g., *build houses* is atelic whereas *build a house* is not), was described by default with a delimited internal argument. While other aspectual features are binary, telicity was assigned a third possible value and annotated as “variable” in the case of degree achievements. This applies to verbs such as *humidifier* ‘moisten’, *lisser* ‘smooth’, *pourrir* ‘rot’, and nouns such as *humidification* ‘moistening’, *lissage* ‘smoothing’, *pourrissement* ‘rotting’, which denote a gradable change of state and exhibit characteristics of both telic and atelic events (Abusch, 1986; Bertinetto & Squartini, 1995; Hay et al., 1999; Rothstein, 2008; a.o.).

As for post-phase, it refers to the possibility for a dynamic eventuality to include a result state (Piñón, 1999; Apothéloz, 2008; Fradin, 2011; Haas & Jugnet, 2013). Post-phase was considered a lexical feature because it determines the distribution of verbs with durative adverbials in a resulting interpretation, as illustrated with *partir* ‘leave’ vs. *arriver* ‘arrive’ in (5).

(5)
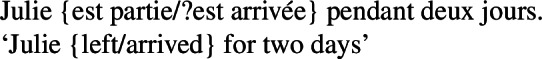

Verbal post-phase was therefore established from the potential result-state reading observed when dynamic verbs are combined with durative adverbials introduced by *pendant* ‘for’. Similarly, nominal post-phase was identified by the possible interpretation as a result state when event nouns are used with the verb *durer* ‘last’, as in (6).

(6)

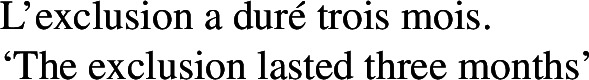

Refined Vendlerian aspectual classes can be distinguished based on the different possible combinations of dynamicity, durativity, telicity and post-phase, as shown in Table 4.

**Table 4:** Features of aspectual classes

Aspectual class	Dynamicity	Durativity	Telicity	Post-phase
State	–	+	–	–
Activity	+	+	–	–
Accomplishment	+	+	+	–
Achievement	+	–	+	–
Degree achievement	+	+	±	–
Left accomplishment	+	+	+	+
Left achievement	+	–	+	+
Left degree achievement	+	+	±	+

#### Semantic role assignment

We analyzed the argument structure of both base verbs and derived nouns by determining the semantic roles that can be assigned to their arguments. Semantic roles were annotated for verbs, eventuality-denoting nouns, as well as certain entity-denoting nouns—typically agent nouns with a patient or a theme argument, as in *l’agresseur de la vieille dame* ‘the old lady’s attacker’ and *le conducteur du véhicule* ‘the vehicle driver’. We used the same set of semantic roles employed to analyze relational types. As with relational types, the identification of semantic roles was based on compliance with the definitions presented in Table 3.

Only arguments that are both semantic and syntactic were taken into consideration. Semantic arguments correspond to participants that are conceptually necessary to the eventuality denoted by the verb or the noun. Syntactic arguments are defined by their syntactic dependency, as evidenced by their incompatibility with the proform *le faire* ‘do it’ in the case of dynamic verbs. For example, the instrument of *trancher* ‘slice’ is a semantic argument—since the action denoted by *trancher* ‘slice’ involves an instrument—but not a syntactic argument (e.g., *Il a tranché la viande, et il l’a fait avec un couperet bien aiguisé* ‘He sliced the meat, and he did it with a sharp cleaver’). Consequently, it was not described in the database.

Nominal argument structures were analyzed based on the ability of derived nouns to combine with the same arguments as their base verbs. The maximum number of syntactic and semantic arguments for each verb and noun was described in the database, regardless of their frequency of co-occurrence. Specifically, extended argument structures were encoded for nominalizations, although all arguments are rarely found together in corpus data. For example, nouns such as *assignation* ‘assignment’ and *insertion* ‘insertion’ were associated with three arguments each—topic, beneficiary and agent for *assignation*; theme, destination and agent for *insertion*—although these nouns are seldom used with the three arguments in the same NPs (as in *l’assignation d’une valeur à une variable par un chercheur* ‘the assignment of a value to a variable by a researcher’, or *l’insertion du CD dans le lecteur par l’auditrice* ‘the insertion of the CD into the player by the listener’).

Verbs allowing for regular syntactic alternations (Levin, 1993) were treated as unique lexical entries, and described according to a conventional pattern. For example, the locative alternation in *X charge Y dans Z* ‘X loads Y into Z’/*X charge Z de Y* ‘X loads Z with Y’ was analyzed following the first syntactic pattern (i.e., X:subject-agent, Y:object-theme, Z:oblique-destination). Anticausatives were encoded as distinct lexical entries due to their non-systematicity and the fact that they may have specific derivatives. As observed by Martin (2010) and Fradin (2016), and as illustrated in (7), morphological doublets ending in -*age* and -*ment* in French can be related specifically to the causative or anticausative constructions of the same verb, highlighting the need to distinguish these constructions for an accurate analysis of base-derivative relationships.

(7)

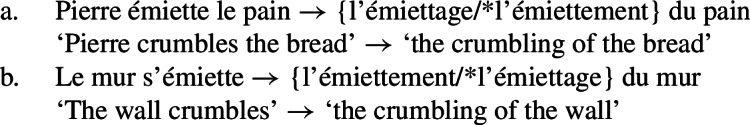

More generally, *se-V* forms were treated as separate lexical entries whenever they instantiated anticausative, intrinsic (e.g., *se méfier* ‘distrust’), autonomous (e.g., *s’apercevoir de* ‘notice’), and autocausative (e.g., *se promener* ‘stroll’) verbs, owing to their lexical idiosyncrasies. By contrast, reflexive (e.g., *se regarder* ‘look at oneself’), mediopassive (e.g., *se manger (avec des baguettes)* ‘be eaten (with chopsticks)’), parallel (e.g., *se rire de* ‘laugh at’), antipassive (e.g., *se saisir de* ‘seize’), and autobenefactive (e.g., *se boire (un verre)* ‘have a drink’) constructions were included in the regular alternation patterns and were not coded as distinct entries.

#### Lexical ambiguity

To account for the ambiguity of deverbal nouns (Rainer, 2014; Ježek, 2007; Melloni, 2011; Lieber, 2018; a.o.), the various meanings of each verb and noun were carefully distinguished and systematically paired in the database. Different meanings of nouns and related verbs were identified based on variations in semantic types, lexical aspect and argument structure, with lexical ambiguity being determined by changes in at least one annotated semantic property. For example, the noun *moulage* ‘molding’ was assigned two separate entries due to differences in semantic type (Event-transposition vs. Artefact-result). Similarly, two eventive meanings of *promotion* ‘promotion’ were distinguished based on aspectual differences, since one meaning refers to an achievement (an advancement in rank or position) and the other to an activity (the act of advertising of something). The noun *révision* ‘revision’ was also associated with two eventive meanings, depending on the role of the internal argument—either a patient, as in *révision de la politique de défense* ‘revision of defence policy’, or a topic, as in *révision du cours d’histoire* ‘review of the history course’.

Nouns were paired with verb meanings that share the most aspectual and role-assignment properties with them, following the principle of nearest semantic proximity, and reflecting the view that derivational processes apply to semantically specified elements (see Mel’čuk, 1993; Fradin & Kerleroux, 2003; Fradin, 2023). Only the verb meanings that are related to derived nouns were described in the database, since the analysis focuses primarily on nouns. According to this descriptive model, different nominal meanings can be associated with the same base verb meaning or with different ones, as illustrated in (8)-(9).

(8)

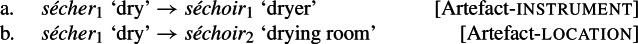

(9)
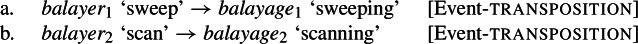

Mixed situations can also be observed, for example when three nominal meanings are related to two verbal meanings, as in (10).^4^

(10)

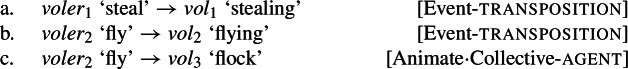

### Annotation procedure

6 team members participated in the semantic annotation of the 5272 selected deverbal nouns. Before proceeding with the full sample analysis, we conducted an initial evaluation of the annotation scheme. 10 samples, each containing 50 verb-noun pairs extracted from the database, were annotated in a double-blind procedure with rotating pairs of annotators. Throughout these training sessions, inter-annotator agreement was evaluated, disagreements were resolved collectively, and the annotation guidelines were further refined. The analysis involved two steps: first, determining the number of distinct meanings associated with each noun, and second, annotating the semantic features for each meaning.

Reliability in the first step was measured using the intraclass correlation coefficient (ICC), which is well-suited for assessing the agreement between annotators evaluating a quantitative feature, such as the number of senses for a word. The raw reliability rate was .63, with an ICC score of .54 across the 10 samples of 50 verb-noun pairs. This result indicates moderate reliability according to Koo and Li’s (2016) scale. Upon closer examination, it appeared that the moderate level of agreement was mostly due to the lack of exhaustiveness when considering the various meanings of a noun. To mitigate the arbitrariness in semantic selection, and since the description was intended to focus on lexicalized meanings, it was decided to rely on lexicographic resources to identify the different possible meanings of the sampled nouns and verbs. Semantic analysis was subsequently performed based on the multiple meanings listed in two reference dictionaries: *Le Petit Robert* (Editions Le Robert, n.d.-a) and *Le Trésor de la Langue Française informatisé* (ATILF - CNRS & Université de Lorraine, n.d.). Corpus occurrences were examined for words that were not present in the dictionaries. In all instances, examples drawn from FRCOW16A or directly from the web were added to the database to illustrate the different nominal and verbal meanings described.

Reliability in the second step, which involved analyzing the semantic features of each verb-noun pair, was assessed using Cohen’s kappa, as well as the prevalence-adjusted bias-adjusted kappa (PABAK) to better account for response probabilities and annotator bias (Byrt et al., 1993). The analysis revealed satisfactory agreement levels, with an overall kappa score of .67 and a PABAK of .79. Detailed agreement scores for each annotated semantic feature are provided in Table 5.^5^

**Table 5:** Inter-annotator agreement for 10 samples of 50 verb-noun pairs

	Observed agreement	Kappa	PABAK
V Transitivity	.93	.80	.88
V Dynamicity	.99	.90	.98
V Durativity	.86	.53	.74
V Telicity	.73	.55	.61
V Post-phase	.83	.44	.61
V Role of subject	.75	.60	.72
V Role of object	.73	.66	.71
V Role of oblique	.84	.44	.82
N Ontological type	.73	.66	.71
N Relational type	.80	.72	.78
N Dynamicity	.99	.94	.98
N Durativity	.91	.83	.87
N Telicity	.87	.79	.83
N Post-phase	.91	.83	.87
N Role of 1st arg.	.67	.58	.64
N Role of 2nd arg.	.81	.64	.79
N Role of 3rd arg.	.97	.39	.95
Average	.84	.67	.79

Once the test phase was completed, the remaining 4772 verb-noun pairs were analyzed by single annotators. Their task was to identify and pair verbal and nominal meanings, and assign values to all relevant properties. This annotation process spanned 2 years, during which the team held weekly meetings to collaboratively resolve challenging cases. After completion of the annotation, 2 team members conducted a final round of homogenization and quality control to ensure consistency across the database. The total time spent on semantic annotation was estimated at 2200 hours. The final version of the database includes 8202 nominal lexemes, paired with 2809 distinct verbal lexemes.

## Results

In this section, we present the semantic content of the database, which results from the annotation of the sampled verb-noun pairs, and use it to address general research questions on nominalizations. We first detail the semantic properties of the nouns and verbs in the database (3.1), before examining the semantic functions of nominalizing processes (3.2), the preservation of lexical aspect and argument structure between verbs and nouns (3.3), and the organization of morphological families formed by deverbal nouns (3.4).

### Nominal and verbal features

As outlined in the previous section, SONDE describes the semantic properties of a large sample of French nominalizations and their base verbs, including their semantic type, lexical aspect, and ability to assign semantic roles to their arguments. This subsection provides detailed information on these semantic features, for both nouns (Sect. 3.1.1) and verbs (Sect. 3.1.2).

#### Nouns

**Fig. 1:**
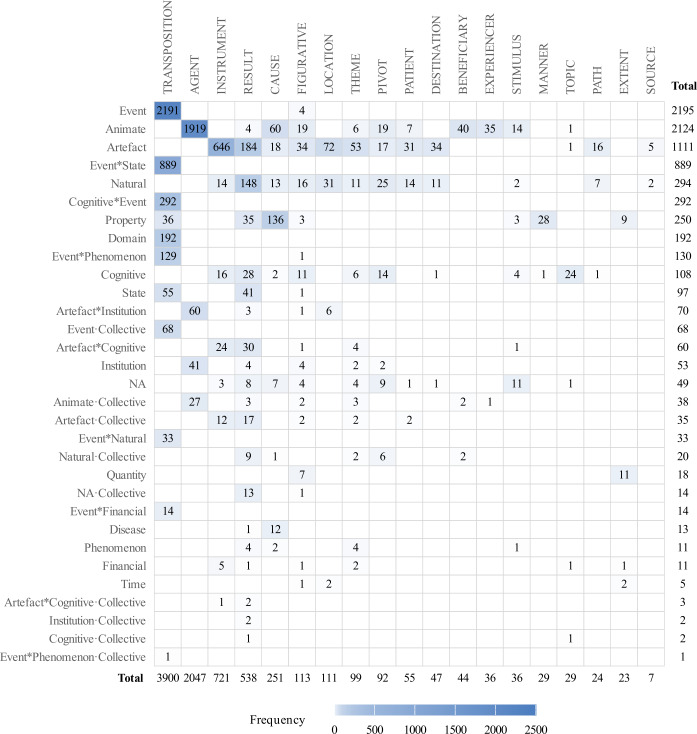
Combinations between ontological and relational types with number of occurrences in SONDE. Ontological and relational types are ordered by decreasing frequency, from top to bottom and from left to right

We begin by examining the distribution of semantic types across the 8202 nominal entries in the database. As shown in Fig. 1, ontological and relational types are unevenly represented in the sample. The most prevalent ontological types are Event (27% of the entries) and Animate (26%), followed by Artefact (14%) and Event*State (10%). These 4 types cover together more than three-quarters of the nouns. At the other end of the spectrum, 12 semantic types (e.g., Quantity, Phenomenon, Time) are each instantiated by 20 nouns (0.25%) or fewer. Unsurprisingly, the distribution of ontological types in deverbal nouns differs clearly from that in simplex nouns, where entity-denoting nouns are dominant and eventuality-denoting nouns are less common. For example, in the sample of 3489 French simplex nouns analyzed by Tribout et al. (2014), 76% denote animate or inanimate entities, 10% events, states or properties, and 14% other ontological types. The distribution of these three categories is significantly different between the two samples ($\chi ^{2}$ (2, N = 11,898) = 1661, *p* < .001). The strong bias of deverbal nouns towards event denotation can be imputed to their morphological relationship with the verbal class. It is consistent with the idea that most event-denoting nouns in a language like French are derived from verbs. However, this referential inclination should not be misinterpreted as implying that deverbal nouns essentially denote events, since a large proportion of them can also refer to animates or artefacts.

As far as relational types are concerned, the dominant types are transposition (48% of the nominal entries), agent (25%) and, to a lesser extent, instrument (9%) and result (7%). By contrast, 10 relational types, such as patient, topic, and path, each have less than 60 instances (0.75%) in the database. The combination between ontological and relational types reflects their skewed distribution in different ways. As can be seen in Fig. 1, only a limited number of combinations between ontological and relational types are instantiated, since 130 (22% of all possible combinations) are actually observed in the database. The most frequent combined types result from the pairing of the most frequent ontological and relational types. These include Event-transposition (27%), Animate-agent (23%), Event*State-transposition (11%), and Artefact-instrument (8%), as exemplified in (11).

(11)
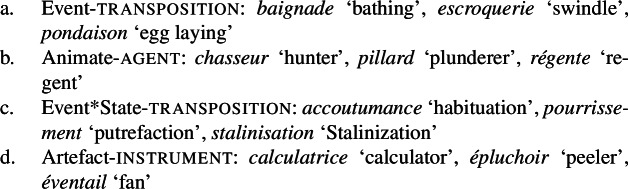

On the other hand, 26 combined types occur only once in the database, for example Phenomenon-stimulus (*senteur* ‘scent’), Animate-topic (*connaissance* ‘acquaintance’), and Cognitive-path (*passage* ‘text passage’).

In some cases, the relationship between ontological and relational types may tend towards exclusivity. This is particularly evident with Agent and animate, since 90% of the animate nouns denote agents and 94% of the agent nouns denote animates. In other cases, exclusivity seems to be one-sided, implying that an ontological or relational type combines mostly with a single relational or ontological type, but not reciprocally. A typical example is the association of Event and transposition, since all event nouns are transpositional (with the exception of figurative meanings), whereas only 56% of the transpositional nouns denote events. However, more often than not, a many-to-many relationship between ontological and relational types can be observed, even for semantic types with a limited number of occurrences. For example, the ontological type Cognitive, represented by 108 occurrences in the database, is associated with 11 different relational types, each of which combines with between 2 and 20 ontological types. Similarly, the relational type theme, instantiated by 99 nouns, is linked to 12 different ontological types, each of which can be paired with 4 to 12 relational types. It remains that, even in these many-to-many configurations, the relative frequency of the combinations is quite variable. The diversity of semantic types, considering both the number and frequency of their combinations, can be estimated using Shannon entropy, which quantifies the uncertainty associated with a probability distribution and reflects the dispersion of semantic information. The entropy of ontological types ranges from 0 (e.g., Event⋅Collective) to 2.81 (Cognitive), while the entropy of relational types ranges from 0.43 (agent) to 2.87 (result). The observed differences reveal considerable variation among semantic types with respect to the diversity of cross-type combinations.

To analyze the aspectual properties of deverbal nouns, we focus on the 4161 nouns (51% of the lexemes) that denote an eventuality, whether their ontological type is Event, State, Property, or any complex type that includes them as a facet. Note that, while nouns denoting events and states were annotated for the 4 aspectual features described in Sect. 2.2.2, nouns denoting properties were only analyzed for dynamicity. Features such as durativity, telicity, and post-phase are not applicable to properties, because properties are atemporal situations and cannot be located in time. As shown in Table 6, the most frequently observed features in the database are [+dynamic], [+durative], [+telic], and [–post-phase]. These features are exemplified by the nouns in (12).

**Table 6:** Distribution of aspectual features across eventuality-denoting nouns

	Dynamicity	Durativity	Telicity	Post-phase
+	3814 (92%)	3044 (73%)	1821 (44%)	542 (13%)
–	347 (8%)	867 (21%)	1495 (36%)	3369 (81%)
±	-	-	595 (14%)	-
NA	-	250 (6%)	250 (6%)	250 (6%)

(12)

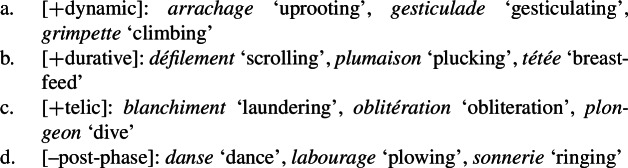

The combination of dynamicity, durativity, telicity, and post-phase forms aspectual classes, whose distribution is presented in Table 7. The most represented classes are activities, accomplishments, and achievements, as illustrated in (13).

**Table 7:** Distribution of aspectual classes across eventuality-denoting nouns (excluding property-denoting nouns)

Aspectual class	Raw count	%		Aspectual class	Raw count	%
Activity	1397	35.7		Left degree achievement	289	7.4
Accomplishment	890	22.8		Left achievement	189	4.8
Achievement	678	17.3		State	97	2.5
Degree achievement	306	7.8		Left accomplishment	64	1.6

(13)

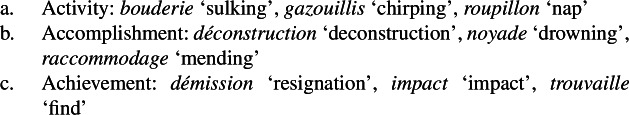

Turning to argument structures, we first consider the maximum number of arguments potentially associated with the nouns included in the database. 46% of the 8202 nominal lexemes are not associated with any argument, while the maximum number of arguments for nouns that license them is preferably 2 (27% of the lexemes), followed by 1 (24%) and then 3 (3%). The nouns with at least one argument mostly denote eventualities (88%), but entity-denoting nouns are not excluded. On the one hand, the denotation of eventualities is not sufficient to ensure the presence of argument structure. For example, some nouns referring to domains (*jardinage* ‘gardening’), weather events (*saucée* ‘downpour’), or collective events (*braderie* ‘street market’) do not license arguments. On the other hand, as mentioned in Sect. 2.2.3, certain entity-denoting nouns can introduce arguments, provided that they involve an underlying event structure (e.g., *l’inventeur de cette machine* ‘the inventor of this machine’, *le destinataire de ce courrier* ‘the recipient of this letter’, *les tenants de cette idéologie* ‘the supporters of this ideology’). Examples of nouns with 1, 2, or 3 arguments are provided in (14).

(14)
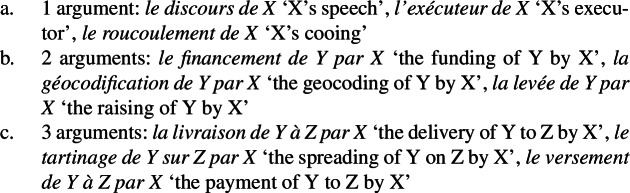

As indicated in Table 8, the most frequent semantic roles assigned to the arguments of deverbal nouns are agent (*la mutinerie des prisonniers* ‘the mutinery of the prisoners’), patient (*le rabotage du budget* ‘the cutting of the budget’), cause (*le clapotis de l’eau* ‘the lapping of the water’), theme (*le lancer du dé* ‘the throwing of the die’), and beneficiary (*l’arrosage de la pelouse* ‘the watering of the lawn’).

**Table 8:** Distribution of semantic roles across nominal arguments

Role	Raw count	%		Role	Raw count	%
Agent	1986	27.7		Destination	160	2.2
Patient	1440	20.1		Stimulus	81	1.1
Cause	861	12.0		Location	34	0.5
Theme	802	11.2		Source	33	0.5
Beneficiary	561	7.8		Path	31	0.4
Topic	419	5.8		Extent	9	0.1
Pivot	302	4.2		Instrument	6	0.1
Result	257	3.6		Manner	2	0.0
Experiencer	191	2.7				

To further investigate the semantic properties of deverbal nouns, we examine their relationships in the database. Fisher’s exact test^6^ shows that all annotated semantic features are significantly associated pairwise (*p* < .001). We use Goodman-Kruskal’s *τ* to further quantify the strength of the relationship between pairs of semantic features. This measure of association offers the advantage of being directional, making it possible to evaluate the unilateral dependence between two semantic features. It is based on random category assignment and measures the percentage improvement in predictability of a categorical variable *y* given another categorical variable *x*. The measure *τ*(*x*,*y*) ranges from 0 when *y* is fully independent from *x* to 1 when *y* is fully dependent on *x*.

**Fig. 2:**
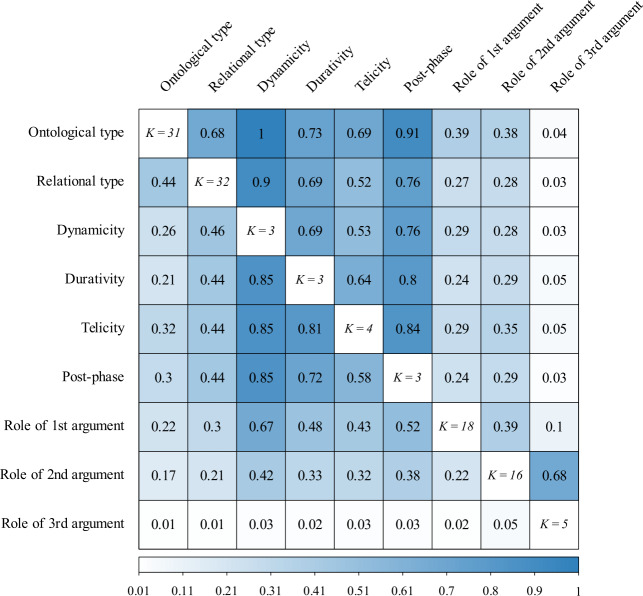
Associations between nominal properties according to Goodman and Kruskal’s *τ*. The values correspond to the directional association measure *τ*(*x*,*y*), where *x* represents the predictor (row) and *y* the response variable (column). The diagonal indicates the number of levels in *x*

Goodman-Kruskal’s *τ* for all pairs of semantic features annotated in SONDE is presented in Fig. 2. Important differences can be observed regarding both the predictive power of the various features and their ability to be predicted. Semantic types emerge as the strongest predictors of other features, with an average *τ* as predictor equal to .60 for ontological types, and .49 for relational types. In contrast, semantic roles show a much weaker predictive power, especially the role of the 3rd argument, which has an average *τ* as predictor of .03. On the other hand, aspectual features are the most accurately predicted, in particular dynamicity and post-phase, as these achieve an average *τ* as response of .70 and .63, respectively. Conversely, ontological types and semantic roles assigned to the 3rd argument are the most difficult features to predict, with an average *τ* as response of .24 and .12, respectively. It appears that aspectual features show a strong overall interpredictability—although this may be influenced by the lack of aspectual properties in entity-denoting derivatives. Aspectual features contrast in that respect with role-assignment properties, whose interdependence appears to be weak in either direction (except in the case of the 2nd argument predicting the 3rd argument).

Additionally, more or less asymmetrical relations can be observed between semantic features. For example, post-phase and dynamicity approach symmetry in interprediction, with *τ* = .85 and *τ* = .76, whereas post-phase and ontological type show a clear asymmetry, with the former being a much better predictor of the latter (*τ* = .91) than the reverse (*τ* = .30). Interestingly, ontological and relational types are asymmetrically associated, since ontological type better predicts relational type (*τ* = .70) than vice versa (*τ* = .44). This pattern reflects the unbalanced relationships between ontological and relational types, as discussed in the case of Event and transposition. It supports not only the distinction between ontological and relational types in the semantic description of deverbal nouns, but also the conclusion that the relational classification—often emphasized in studies of nominalization—is insufficient to fully account for the semantic properties of deverbal nouns.

#### Verbs

The description of verbs in SONDE differs from that of nouns in that it is not intended to be semantically exhaustive and focuses exclusively on verb meanings related to derived nouns. Nevertheless, for information purposes, we detail in this section the properties of the 2809 verbal lexemes included in the database. The distribution of their aspectual features is presented in Table 9. As in the case of nouns, the dominant features are [+dynamic], [+durative], [+telic], and [–post-phase], for which examples are provided in (15).

**Table 9:** Distribution of aspectual features across verbs

	Dynamicity	Durativity	Telicity	Post-phase
+	2669 (95%)	2098 (75%)	1406 (50%)	509 (18%)
–	140 (5%)	711 (25%)	955 (34%)	2300 (82%)
±	-	-	448 (16%)	-

(15)
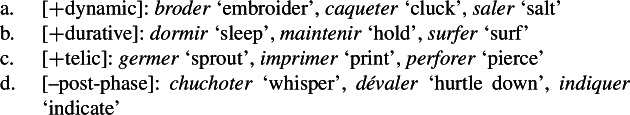

The distribution of aspectual classes is shown in Table 10. Again, the results align with those observed for nouns, with activities (*pleurnicher* ‘whine’), accomplishments (*récapituler* ‘summarize’) and achievements (*abdiquer* ‘abdicate’) being the most prevalent classes, covering together 70% of the verbs.

**Table 10:** Distribution of aspectual classes across verbs

Aspectual class	Raw count	%		Aspectual class	Raw count	%
Activity	815	29.0		Degree achievement	200	7.1
Accomplishment	620	22.1		Left achievement	186	6.6
Achievement	525	18.7		State	140	5.0
Left degree achievement	248	8.8		Left accomplishment	75	2.7

Regarding the number of arguments, verbs with 2 arguments are by far the most common (66% of the verbs), followed by verbs with 1 argument (27%), and then verbs with 3 arguments (7%). One weather verb (*grêler* ‘hail’) is present in the database and described as having no argument. Various combinations of syntactic functions can be observed: 27% of the verbs have only a subject (*X pétille* ‘X fizzes’), 56% involve a subject and an object but no oblique (*X ratisse Y* ‘X rakes Y’), 10% have a subject and an oblique but no object (*X adhère à Y* ‘X adheres to Y’), and 7% include a subject, an object and an oblique (*X accole Y à Z* ‘X joins Y to Z’).

Table 11 shows the distribution of semantic roles across verbal arguments. Similar to nominal arguments, the most frequently observed semantic roles for verbal arguments are agent, patient, cause, theme, and beneficiary. When considering the mapping between semantic roles and syntactic functions, 126 distinct valency patterns can be identified in the database. The most frequently observed patterns are transitive verbs with a patient object and an agent subject (16a) or a cause subject (16b), each accounting for 12% of the verbs, followed by intransitive verbs with a patient subject (10%) as in (16c), transitive verbs with an agent subject and a beneficiary object (9%) as in (16d), and intransitive verbs with an agent subject (9%) as in (16e).

**Table 11:** Distribution of semantic roles across verbal arguments

Role	Raw count	%		Role	Raw count	%
Agent	1511	29.8		Destination	116	2.3
Patient	1020	20.1		Stimulus	96	1.9
Cause	619	12.2		Source	31	0.6
Theme	477	9.4		Location	29	0.6
Beneficiary	369	7.3		Path	23	0.5
Topic	302	6.0		Extent	10	0.2
Result	165	3.3		Manner	7	0.1
Experiencer	158	3.1		Instrument	4	0.1
Pivot	131	2.6

(16)

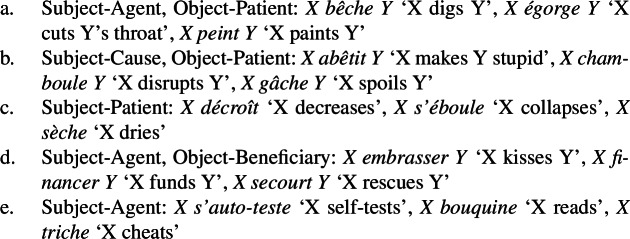

**Fig. 3:**
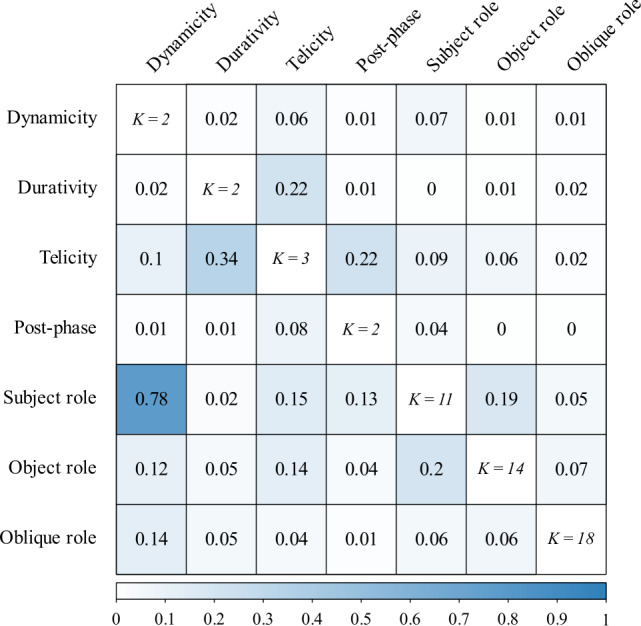
Associations between verbal properties according to Goodman and Kruskal’s *τ*. The values correspond to the directional association measure *τ*(*x*,*y*), where *x* represents the predictor (row) and *y* the response variable (column). The diagonal indicates the number of levels in *x*

As in the case of nouns, we examine the relationships between the semantic properties annotated for verbs, focusing on the 4 aspectual features and the semantic roles of the subject, object and oblique arguments of the verbs. According to Fisher’s exact test, all semantic features are significantly associated pairwise (*p* < .01). The strength of the unilateral associations between pairs of features is assessed using Goodman-Kruskal’s *τ*. As reported in Fig. 3, the *τ* values for verbs are notably lower than those obtained for nouns, with only 2 pairs of verbal properties showing a score equal to or greater than .30. The interdependence between semantic features appears to be weaker in the verbal domain than in the nominal domain, which may be explained by the fact that aspectual and role-assignment properties apply only to certain nouns, whereas they concern all verbs. Non-relevant categories in the nominal domain are closely interrelated, making the relationships between semantic properties more systematic than in the verbal domain.

Most verbal features provide limited information about the other features, as seen with post-phase and dynamicity, which have an average *τ* as predictor of .02 and .03, respectively. Others may be more informative, but only with respect to specific features, as in the case of subject role predicting dynamicity and, to a lesser extent, telicity predicting durativity and post-phase. Conversely, many properties are difficult to predict, such as oblique and object roles, which have an average *τ* as response of .03 and .06, respectively. While most predictions are roughly symmetrical (with generally low *τ* values), a few are clearly asymmetrical, as observed with dynamicity and semantic roles. In particular, the strong asymmetrical association between dynamicity and subject role can be attributed to the fact that a large proportion of verbs in the database have an agent subject and that all these verbs are dynamic, whereas the subjects of dynamic verbs can be assigned 10 different semantic roles.

### The semantics of deverbal processes

In this section, we investigate the semantic properties of the various morphological processes used to derive nouns from verbs in French. The main research questions concern the diversity of the processes, their specific semantic functions, and the extent of their differences. Unsurprisingly, a many-to-many relationship is observed between form and meaning in verb-to-noun derivation. We first examine the polyfunctionality of deverbal processes, focusing on how a single process can convey multiple meanings (Sect. 3.2.1). Next, we explore the semantic similarity between processes, depending on how the same meanings can be expressed by different processes (Sect. 3.2.2).

Note that, in these analyses, we do not take into consideration the figurative nominal meanings which are not related to verbs, as they may not be informative about the semantics of derivational processes—113 nominal lexemes were thus disregarded in the analysis. Furthermore, we postulated that a derivational semantic function can be identified when at least two nouns derived through the same process instantiate the same combined semantic type (i.e., the same pairing of an ontological and a relational type). Semantic types that occur only once for a given process may be idiosyncratic lexicalized meanings rather than products of derivation, and were excluded from the analyses by default. This applies to 369 nominal lexemes, in addition to the figurative ones. Finally, our analysis of the semantic properties of deverbal processes was conducted on a subset of SONDE that comprises 7720 nominal lexemes.

#### Polyfunctionality

Most deverbal processes appear to be polyfunctional, in the sense that they have multiple semantic functions and can assign different semantic types to the words they form. It is important to distinguish polyfunctionality, defined as process ambiguity, from lexical ambiguity, which refers to the number of meanings words can have. The derivatives produced by a polyfunctional process very rarely instantiate all its semantic functions. For example, the suffix -*ance* is associated with 13 distinct functions in our dataset, while the highest number of meanings observed for a deverbal noun in -*ance* is 5.

**Fig. 4:**
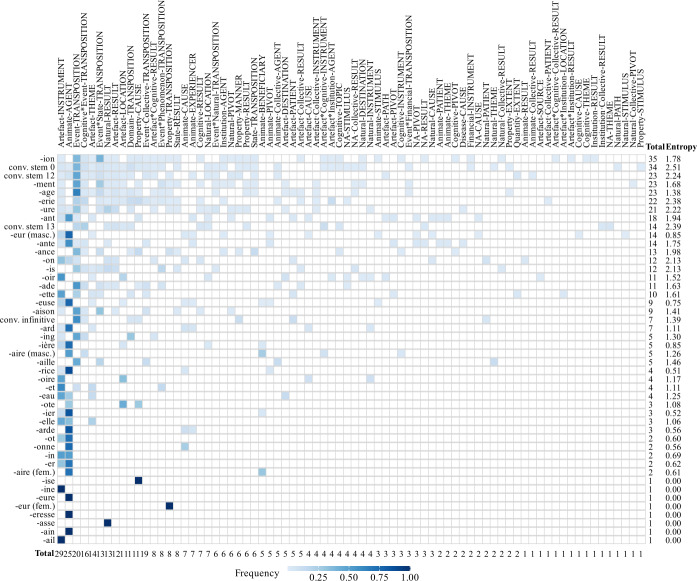
Semantic functions instantiated by morphological processes. Colors represent the distribution of derivatives across functions for each process. Processes are ordered from top to bottom by decreasing number of functions. Functions are ordered from left to right by decreasing number of processes. The total number of functions per process and their entropy are indicated on the right of the figure. The total number of processes per function is indicated at the bottom of the figure

In SONDE, the number of functions served by deverbal processes varies significantly, ranging from 1 (e.g., for -*ail*, -*asse*, and -*ise*) to 35 (for -*ion*), with an average of 8.9 functions per process (*SD* = 8.8). Moreover, as shown in Fig. 4, functions are not evenly distributed across the derivatives of a polyfunctional process. For instance, the semantic type Animate-agent (*chasseur* ‘hunter’) is highly represented among the masculine derivatives in -*eur*, with 803 occurrences in the dataset, whereas Artefact-instrument (*batteur* ‘whisk’) is less frequent, with 174 occurrences, and Cognitive-cause (*révélateur* ‘indicator’) is marginal, with 2 occurrences only. Additionally, some processes seem to be more balanced than others in terms of function frequency. For example, while both -*aison* and -*euse* exhibit 9 functions each, the distribution of their derivatives per function differs considerably, ranging from 2% to 48% for -*aison*, and from less than 1% to 76% for -*euse*, with distinct standard deviations in percentage of derivatives per function (17 and 25, respectively). To provide an accurate assessment of the semantic diversity of the processes, we calculated their entropy, which takes into account both the number and the frequency of functions associated with each process (see Sect. 3.1.1). As shown in Fig. 4, the most diverse processes are not necessarily those with the greatest number of functions. This is evident as conversion 0, conversion 13, and -*erie* exhibit the highest entropy while ranking 2nd, 5th, and 11th in function count. Conversely, highly productive suffixes such as -*ion*, -*age*, and -*ment* hold top positions for the number of functions (1st, 3rd, and 4th, respectively) but rank 10th, 19th, and 12th in entropy. Conversion, in particular, appears to exhibit the greatest balance between a high number of functions and an even distribution across derivatives, with conversions from stems 0, 12 and 13 ranking among the 4 most diverse deverbal processes.

Based on the information included in SONDE, we further examined the foundations of polyfunctionality in deverbal processes. A straightforward explanation for the polyfunctionality of deverbal processes is the disparity between the limited inventory of available processes and the large number of semantic functions that need to be expressed. In terms of systemic economy, this contrast alone justifies the existence of polyfunctionality. However, such an explanation implies a static view of the number of available forms, meanings to express, and their interrelations, which may not be fully adequate. First, the number of derivational forms in a language is neither fixed nor predetermined: it can expand with the introduction of new processes, for example through morphological borrowing (Gardani et al., 2015). Second, an efficient distribution of functions across processes would ideally be based on complementary classes. With 76 functions and 46 processes, this would result in an average of 1.7 functions per process, far from the 8.9 observed in our database. In reality, many semantic functions are common to multiple deverbal processes, which can additionally cause competition—an effect that seems undesirable from a systemic perspective (see Sect. 3.2.2). Third, the simple need to balance meanings and forms does not account for the dynamics of meaning, which involves potential semantic extension driven by conceptual relations and semantic change over time. Taken together, these observations suggest that polyfunctionality may be motivated semantically and that the associations between semantic functions in deverbal processes may not be arbitrary.

To analyze these associations, we proceeded in two steps. First, we aimed to identify positively associated functions within the derivational system. To do so, we applied Fisher’s exact test to every pair of functions instantiated by at least one process in the dataset. Out of the 2850 possible pairs, 1516 are actually observed, with the number of processes realizing them ranging from 1 to 15. Notably, more than half of these pairs (53%) are observed for only 1 process, and 85% are observed for 3 processes or fewer. Among these 1516 pairs, 384 (25%) show a significant positive association (*p*<.05, odds ratio >1).^7^ For example, Fisher’s exact test revealed that the functions Event-transposition and Artefact-result are positively associated (*p*<.001, with an infinite odds ratio).

In the second step, we investigated potential semantic extensions from one function to another. To this end, we compared the conditional probabilities of $F_{1} $ given $F_{2} $ and $F_{2} $ given $F_{1} $ for each non-random pair, again using Fisher’s exact test. We identified 56 associations (30% of the 384 non-random pairs) in which the conditional probabilities differ significantly from each other. In the case of Event-transposition and Artefact-result, for example, we found that the probability of Event-transposition given Artefact-result is 1, whereas the probability of Artefact-result given Event-transposition is .6. This asymmetry is significant considering the number of processes involved (*p*<.05), which indicates a directional relationship between the two functions. The presence of Event-transposition is required for Artefact-result to occur, but not vice versa, suggesting a semantic extension from the first to the second.

**Fig. 5:**
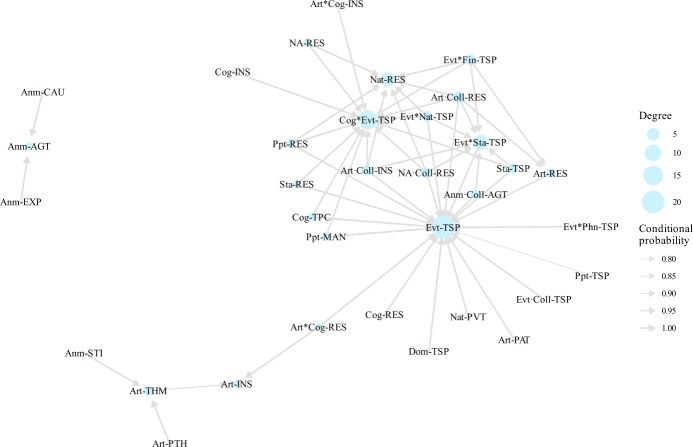
Network of significant, positive, and directed associations between semantic functions. Node size represents the total number of relationships (incoming and outgoing) associated with a semantic function. Edge thickness represents the conditional probability of one function occurring given the presence of another. Abbreviations are listed in Tables 2 and 3

Interestingly, certain semantic functions, such as Event-transposition and Animate-agent, appear in multiple associations. To provide a comprehensive overview of the non-arbitrary associations between semantic functions, we employed a network representation (see Fig. 5). Specifically, we constructed a network that includes only the 56 unidirectional associations for which the probability of $F_{1} $ given $F_{2} $ is significantly greater or smaller than the probability of $F_{2} $ given $F_{1} $. The nodes in the network represent the 34 semantic functions involved in these associations, while the 56 edges between the nodes reflect the strongest conditional probabilities. For instance, we retained the edge Artefact-result → Event-transposition because the probability of Event-transposition given Artefact-result is significantly higher than the reverse probability.

A number of observations can be drawn from Fig. 5. Not all semantic functions play an equally important role within the network. Functions that emit the most edges—those whose realization depends on the presence of other functions and that are potentially generated through semantic extensions—are often associated with collective dimensions or complex semantic types, typically including an entity-denoting facet. Among these functions are Artefact.Collective-result (5 outgoing edges), Artefact.Collective-instrument (4), NA.Collective-result (4), Event*Natural-transposition (4), and Event*Financial-transposition (4). In contrast, functions that receive the most edges—those necessary for the realization of other functions and presumably the source of semantic extensions—are predominantly event-related: Event-transposition (20 incoming edges), Cognitive*Event-transposition (14), and Event*State-transposition (7). A dense network of ontologically diverse but mostly transpositional and resultative meanings is organized around these eventive functions, which supports the pivotal role of action meanings in the semantic structure of deverbal processes. Only two parts of the network are not connected to eventive functions: one centered on animates (around Animate-agent) and the other on artefacts (around Artefact-instrument).

While the importance of semantic functions can be examined at a local level, it can also be assessed across the entire network using the degree centralization score. This metric quantifies the degree to which a graph depends on its most central nodes. In our analysis, we obtain a degree centralization score of .261. A Conditional Uniform Graph (CUG) test (Butts, 2008), which evaluates whether a specific characteristic of a graph is statistically significant, reveals that the centralization score for our graph is consistently higher than the values observed in 2000 simulated networks sharing the same number of vertices and density (defined as the ratio of the actual number of edges to the maximum possible number of edges in the network). These results confirm that the observed level of centralization is statistically significant, highlighting the highly centralized and radial nature of the network. Given that the most important functions in the network are predominantly event-related, these findings support the idea that the morphological relation to the verb shapes the semantics of deverbal nouns, fundamentally distinguishing them from other nominal categories, such as simplex, denominal, and deadjectival nouns.

#### Similarity

As can be seen in Fig. 4, semantic functions are unevenly distributed across deverbal processes. While 16 functions are only represented by 1 process (e.g., Artefact-source for -*oir*, as in *plongeoir* ‘diving board’), most functions are common to multiple processes, in variable proportions. Functions such as Animate-theme (*habitant* ‘inhabitant’), Disease-cause (*tremblante* ‘scrapie’) and Quantity-extent (*pincée* ‘pinch’) are common to 2 processes, while at the other end of the spectrum, Event-transposition (*floraison* ‘blossoming’), Animate-agent (*poursuivant* ‘pursuer’) and Artefact-instrument (*faucheuse* ‘harvester’) are shared by 20, 25 and 29 processes, respectively. On average, semantic functions are realized by 5.4 processes (*SD* = 5.3). These shared functionalities result in semantic similarity between processes to varying degrees. While some pairs of processes may serve the exact same functions (e.g., -*eure* and -*eresse* which exclusively form Animate-agent derivatives), most of them differ in their functional capacities (e.g., -*age* and -*ment* which are associated with 23 functions each, but have only 17 in common).

**Fig. 6:**
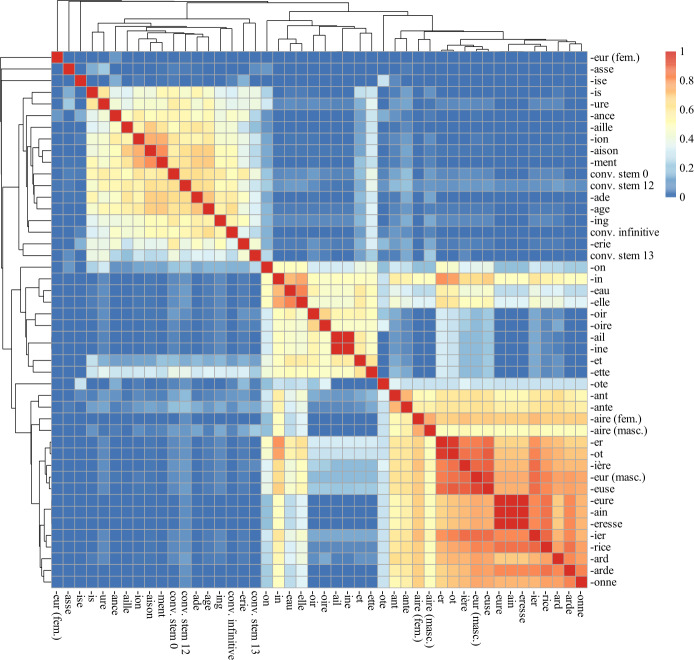
Similarity between morphological processes based on shared semantic functions. Warm colors indicate a strong similarity

As discussed by Salvadori et al. (2024), various methods can be used to estimate the semantic similarity between derivational processes. These methods are based either on the proportion of shared and unshared functions between two processes (richness measures) or on the number of their derivatives that instantiate shared and unshared functions (abundance measures). To assess the similarity between deverbal processes while accounting for the important variation observed in the function distribution of each process, we opted for an abundance measure. Specifically, we used percentage similarity (*PS)*, which compares the realization frequencies of shared functions between processes.^8^ We applied this measure to normalized data, given the partly non-random sampling method and the overrepresentation of weakly productive suffixes in the database. The results are presented in Fig. 6 as a heatmap, which also includes a hierarchical clustering using the average method to group processes according to their similarity.

Leaving aside the suffixes -*eur* (feminine), -*asse* and -*ise*, which are monofunctional and produce very few deverbal nouns (together accounting for only 0.08% of the data), 3 main clusters of deverbal processes can be identified based on semantic similarity. The processes included in each cluster are listed in Table 12. Additionally, a hierarchy can be observed among these three groups, as Clusters 2 and 3 form a higher-level grouping that contrasts with Cluster 1. A total of 4788 nouns are derived from the processes in Cluster 1, 401 from those in Cluster 2, and 2525 from those in Cluster 3. Table 13 presents the 5 main semantic types instantiated by the nouns in each cluster, with some clearly dominant semantic types, such as Event-transposition in Cluster 1, Artefact-instrument in Cluster 2, and Animate-agent in Cluster 3. Overall, the clustering appears to be primarily driven by ontological description, as a clear distinction emerges between processes that produce mainly nouns denoting eventualities (4016 nouns, 84% in Cluster 1), inanimate entities (324 nouns, 81% in Cluster 2), and animate entities (2030 nouns, 80% in Cluster 3). These patterns of semantic similarity not only confirm the importance of ontological description in the semantics of deverbal processes, but also show the preference of processes for distinct broad categories of meaning. Further, they reveal the existence of converging polyfunctionalities, since similarity clusters are organized around consistent groups of functions shared between polyfunctional processes.

**Table 12:** Main clusters of morphological processes based on semantic similarity

	Morphological processes
Cluster 1	-*is*, -*ure*, -*ance*, -*aille*, -*ion*, -*aison*, -*ment*, conversion 0, conversion 12, -*ade*, -*age*, -*ing*, conversion infinitive, -*erie*, conversion 13
Cluster 2	-*on*, -*in*, -*eau*, -*elle*, -*oir*, -*oire*, -*ail*, -*ine*, -*et*, -*ette*
Cluster 3	-*ote*, -*ant*, -*ante*, -*aire* masc, -*aire* fem, -*er*, -*ot*, -*ière*, -*eur* masc, -*euse*, -*eure*, -*ain*, -*eresse*, -*ier*, -*rice*, -*ard*, -*arde*, -*onne*

**Table 13:** Most frequent semantic types in process clusters

Cluster 1	Cluster 2	Cluster 3
Event-transposition (45%)	Artefact-instrument (49%)	Animate-agent (74%)
Event*State-transposition (18%)	Artefact-location (9%)	Artefact-instrument (14%)
Cognitive*Event-transposition (6%)	Event-transposition (7%)	Animate-cause (2%)
Domain-transposition (4%)	Animate-agent (5%)	Animate-beneficiary (1%)
Artefact-result (4%)	Artefact-theme (3%)	Animate-experiencer (1%)

Semantic similarity is a primary cause of morphological competition. Different derivational processes can compete in the formation of new words if they produce words with similar semantic types, particularly when these processes are highly productive. As discussed in the literature, morphological competition can be resolved by a variety of factors, including phonological, morphological, semantic, syntactic, stylistic, and sociolinguistic ones (see, e.g., Plag, 1999; Lindsay & Aronoff, 2013; Naccarato, 2019; Schirakowski, 2020; Guz, 2009; Säily, 2011). However, the reasons behind the emergence of similarity can be questioned, and one may ask why identical semantic types can be produced by multiple processes. From a systemic perspective, semantic similarity is not economical—an optimal distribution of meanings across forms would rather be complementary. Unlike polyfunctionality, similarity in morphological processes is not required by the imbalance between a limited number of forms and a larger number of meanings; it may have distinct underlying causes. While multiple factors such as diachronic change and linguistic contact can determine similarity, it can also be hypothesized that the existence of shared semantic functions between morphological processes depends, at least to some extent, on semantic associations between functions. The semantic relatedness between functions may be one of the reasons why a process expands its functionality and potentially competes with other processes. In other words, polyfunctionality—when semantically motivated—may be a factor that contributes to similarity.

To investigate this potential contribution, we analyzed the relationship between the number of functions shared between two processes and the proportion of semantically motivated functions among shared functions. Specifically, we used an index of motivated similarity, defined as the proportion of shared functions that result from semantic extension.^9^ This index ranges from 0 to 1, depending on whether none or all of the shared functions arise from semantic extension within each process. The relevant patterns of semantic extension are those identified in the analysis of polyfunctionality (see Sect. 3.2.1). We used a Poisson regression to predict the number of shared functions between two processes based on the motivated similarity index. The results of this analysis reveal that semantic extensions among functions significantly increase the number of shared functions (Rate ratio = 12.2, *p* < .001, Deviance explained = .56). The more shared functions result from semantic extension, the greater their number. Accordingly, the conceptual associations that drive polyfunctionality, and semantic extensions in particular, can be seen as a cause of semantic similarity. Such an influence is apparent in diachrony. For example, the French suffix -*age*, originally used to create nouns that denote taxes on certain activities, expanded its semantic functions by metonymy to form words that refer to the activities themselves (Baldinger, 1950; Fleischmann, 1976; Rainer, 2005). Due to this semantic extension, it entered competition with suffixes such as -*ment*, which were already available for forming event-denoting nouns (Uth, 2010). We observe the same phenomenon in synchrony, as semantic extensions stem from conceptual associations that can develop independently of the semasiological system in which they occur, potentially leading to morphological competition.

### Semantic preservation

In this section, we analyze how the aspectual and argumental properties of base verbs are preserved or affected by the nominalization process. Cases of non-preservation are examined to determine whether they are caused by identifiable factors. We first investigate the preservation of lexical aspect between verbs and nouns (Sect. 3.3.1), then assess whether arguments and their semantic roles are maintained in derived nouns compared to their base verbs (Sect. 3.3.2).

#### Lexical aspect

It is often implicitly assumed that eventuality-denoting nominalizations inherit the lexical aspect of their base verbs. The idea of a cross-categorial transfer of aspect from verbs to nouns has been explicitly formulated by authors such as Gross and Kiefer (1995, 51) and Melloni (2006, 288), and it has been defined by Fábregas et al. (2012) as the Aspect Preservation Hypothesis (APH). In its most nuanced fomulation, the APH states that “the aspectual information of a deverbal nominalization is built from the space of possibilities that the base verb allows” (Fábregas & Marín, 2017, 157). The APH postulates that nominalization cannot give rise to new aspectual information that is not already available in the semantics of the base verb. For instance, a deverbal noun could only denote a state provided that its base contains a state component. The basic assumption behind aspectual preservation is that aspect is primarily a verbal property, and all aspectual information in the nominal domain originates from the verbal domain.

Before examining aspectual inheritance between verbs and nouns in SONDE, it is important to note that the question of lexical preservation is biased by structural differences in lexical aspect between the verbal and nominal domains. Due to their inherent ability to reify reference, nouns include lexical information about eventualities that may be absent from the meaning of verbs. In the case of dynamic eventualities, nouns lexically encode the ability to denote individuated vs. non-individuated actions, through the mass/count distinction.^10^ This is captured at the ontological level by the distinction between Event and Domain, and it relates to the denotation of spatio-temporal occurrences of actions, with count nouns denoting individuated events (e.g., *réunion* ‘meeting’), while mass nouns refer to domains of activity (e.g., *jardinage* ‘gardening’). In the case of stative eventualities, nouns can lexically distinguish between stage-level and individual-level predication, through differences in temporal specification. This is reflected at the ontological level by the distinction between State (e.g., *agacement* ‘irritation’) and Property (e.g., *jugeote* ‘gumption’). While states are episodic eventualities that can be located and delimited in time, properties lack temporal anchoring and represent inherent characteristics of entities. Considering these differences in the light of aspectual preservation, the distinction between Event and Domain could be related to an additional feature of lexical aspect (‘occurrentiality’) that would be applicable in the nominal domain only. Being absent from the verbal domain, such a feature is not involved in verb-to-noun preservation strictly speaking, although it might challenge the idea that the aspectual properties of nouns are necessarily issued from verbs. By contrast, the distinction between State and Property implies a possible reduction in aspectual features, because the semantic categories of duration, telicity and post-phase do not apply to properties. At the very least, lexical preservation should be examined for verbal features that are also relevant to nouns, and when investigating the preservation of lexical aspect, we concentrated on the properties shared by verbs and nouns. Accordingly, property-denoting nouns were only analyzed with respect to the preservation of dynamicity, whereas all aspectual features were considered for other types of eventualities.

We focused on the 4152 eventuality-denoting nouns in SONDE that are not figurative—since figurative meanings are not relevant when investigating the influence of derivation on aspectual preservation. 587 out of these 4152 nouns (14%) do not preserve the lexical aspect of their base. Preservation varies across aspectual features, as shown in Table 14, with telicity being the least preserved feature. The most frequent cases of discrepancies for each feature are exemplified in (17).

**Table 14:** Preservation of aspectual features from base verbs in eventuality nominalizations

	Dynamicity	Durativity	Telicity	Post-phase
Preservation	3914 (94.3%)	3766 (96.4%)	3650 (93.5%)	3793 (97.1%)
Verb + Noun –	237 (5.7%)	26 (0.7%)	194 (5.0%)	111 (2.8%)
Verb – Noun +	1 (0.0%)	113 (2.9%)	21 (0.5%)	1 (0.0%)
Verb ± Noun +	-	-	11 (0.3%)	-
Verb ± Noun –	-	-	27 (0.7%)	-
Verb – Noun ±	-	-	0	-
Verb + Noun ±	-	-	2 (0.1%)	-

(17)
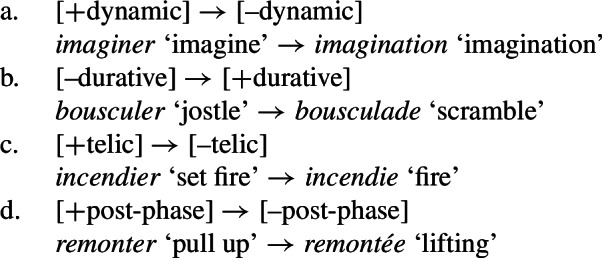

These results confirm the findings of previous empirical studies on nominalizations, which also noted the existence of aspectual discrepancies between bases and derivatives. For example, Balvet et al. (2011) reported aspectual mismatches for 147 out of 639 verb-noun pairs (23%) present in the Nomage resource, based on corpus annotation of nominalizations in French. These discrepancies apparently contradict the hypothesis of aspectual preservation. However, considering that preservation is largely dominant, it can be asked whether aspectual differences between verbs and nouns are idiosyncratic and reflect the effects of lexicalization rather than morphological derivation, in which case they would not violate the APH.

**Fig. 7:**
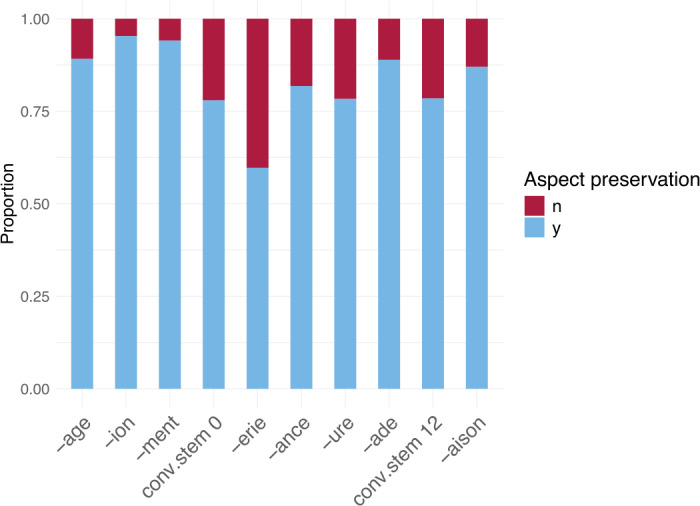
Aspect preservation in the 10 most frequent processes forming eventuality nouns. Processes are ordered from left to right by decreasing frequency

To refine the analysis, we closely examined the relationship between aspectual preservation and specific properties of derivational patterns, namely the formal process involved (suffixation or conversion types) and the semantic properties of the bases. Chi-squared tests were conducted to assess the association of aspectual preservation with morphological processes, aspectual features, and the role-assignment properties of the base verbs. These tests revealed significant relationships (*p* < .05), although the effect sizes were negligible to moderate, with Cramér’s *V* ranging from .06 to .34 (*M* = .12, *SD* = .09). The strongest association was observed with morphological processes. As illustrated in Fig. 7 for the 10 most frequent processes in eventuality-denoting nominalizations (which account for 93% of them), rates of aspectual preservation vary depending on the process. Suffixation in -*erie* and conversion 0 exhibit the highest proportion of non-preserving nominalizations (40% and 22%, respectively), while suffixations in -*ion* and -*ment* appear to be the most preserving processes (5% and 6% of non-preservation, respectively).

To investigate the properties of derivational patterns as joint predictors of aspectual discrepancies, we performed a classification analysis. We used a gradient boosting tree classifier, a learning algorithm that improves decision trees sequentially and has proven effective in morphological tasks (Guzmán Naranjo & Bonami, 2021, 2023). We trained the classifier to predict aspectual preservation based on derivational processes and the semantic properties of base verbs. We applied 10-fold cross validation, and aggregated the results of the 10 models to determine an overall prediction. The classifier achieved a final accuracy of .870, only marginally higher than the No Information Rate baseline of .859 (i.e., the accuracy obtained by always predicting the largest class). This improvement was nevertheless statistically significant according to a binomial test (*p* = .02).

**Fig. 8:**
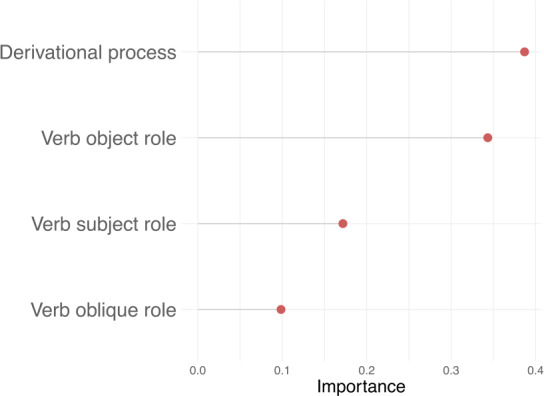
Variable importance for predicting aspectual shifts in gradient boosting tree models

To further explore the conditions of non-preservation, we focused on aspectual discrepancies and examined the shifts in aspectual classes between base verbs and derived nouns. The 10 most frequent shifts (representing 79% of the cases) are presented in Table 15. We analyzed the relationship between these aspectual shifts and both morphological processes and the role-assignment properties of base verbs—excluding aspectual features as they contribute to the definition of aspectual shift categories. Separate chi-squared tests indicated significant associations between the variables (*p* < .001), with a moderate effect size (Cramér’s *V* = [.24,.36], *M* = .33, *SD* = .06). The strongest association was found for morphological processes. As in the analysis of preservation vs. non-preservation, we trained a gradient boosting tree model to determine the predictability of aspectual shifts, based on morphological processes and the argument structure of base verbs. The results showed a relatively low accuracy (.486) which was however significantly higher than the baseline (.222) (*p* < .001). An assessment of predictor importance, based on measures aggregated from 10-fold cross validation, revealed that morphological processes and the semantic roles of verb objects are the most influential variables in predicting aspectual shifts (see Fig. 8).

**Table 15:** Most frequent aspectual shifts between base verbs and derived nouns

Verbal class	Nominal class	Frequency	Example
Accomplishment	Activity	105	*imprimer* ‘print’ → *imprimerie* ‘printing’
Activity	Property	103	*se retenir* ‘hold back’ → *retenue* ‘restraint’
Achievement	Activity	69	*cueillir* ‘pick’ → *cueillette* ‘picking’
Accomplishment	Property	36	*former* ‘form’ → *format* ‘format’
Achievement	Property	36	*croquer* ‘crunch’ → *croquant* ‘crunchiness’
Left accomplishment	Accomplishment	36	*dérouler* ‘roll out’ → *déroulement* ‘rolling out’
Activity	State	25	*amuser* ‘amuse’ → *amusement* ‘amusement’
Left achievement	Achievement	25	*éteindre* ‘switch off’ → *éteignage* ‘switching off’
Achievement	Accomplishment	20	*attenter* ‘attempt’ → *attentat* ‘attack’
Degree achievement	Activity	18	*bronzer* ‘tan’ → *bronzette* ‘sunbathing’

These findings suggest that, although lexical aspect is mostly preserved in eventuality nominalizations, aspectual shifts do occur and cannot be viewed simply as random opacification caused by lexicalization. Derivation can affect lexical aspect, not only by obliterating it in entity-denoting nouns, but also by altering specific features in eventuality-denoting nouns. Aspectual modification is influenced by various properties of derivational patterns, including the semantic properties of the base verbs and the formal process involved. Further evidence for this influence comes from nonce words, which may also exhibit aspectual discrepancies despite not having undergone lexicalization. Previous investigations on deverbal neologisms ending in -*age*, -*ion* and -*ment* have identified distinct trends in aspectual shifts depending on the suffix (Huyghe et al., 2023). The analysis of a random sample of 300 neologisms formed with these suffixes showed that 15% of them exhibit aspectual mismatches with their base verbs. The three suffixes differ significantly both in their tendency to change lexical aspect and in the specific types of aspectual shifts they induce. These results confirm that derivation does not always preserve lexical aspect and can contribute to the development of aspectual properties within the nominal domain.

#### Argument structure

Variation in argument structure has been widely discussed in syntactic studies on nominalizations. A key distinction has been proposed between ‘complex event nominals’ or ‘argument-structure nominals’ on one hand, and ‘result nominals’ or ‘referential nominals’ on the other hand, depending on whether they instantiate argument structure (Grimshaw, 1990; Alexiadou, 2001; Borer, 2003; a.o.). This distinction is characterized by additional properties such as the ability to assign semantic roles, the possibility of an event reading, and compatibility with agent-oriented modifiers and aspectual modifiers—features that apply to complex event nominals but not to result nominals. However, the role of the lexicon in this distinction remains uncertain and underexplored in syntactic research, as the focus is primarily on syntactic structures and contextual realization, and many examples used to illustrate the distinction involve the same nouns appearing in both constructions (e.g., *examination*, *collection*, *replacement*).

From the lexical perspective adopted in SONDE, argument structures are associated with specific noun meanings. Lexemes are analyzed according to their capacity to select arguments and to assign semantic roles, with maximal argument structures described for each meaning. This analysis is conducted independently of the frequency of co-occurrence of arguments in context, as determination and anaphora in the nominal domain often allow arguments to remain unexpressed in NPs. Based on this lexical description, we can ask whether verbal arguments are preserved in derived nouns, as we did for lexical aspect, under the default hypothesis that eventuality nominalizations inherit the argument structure of their base verbs. However, an important difference with aspect is that not only eventuality-denoting nouns, but also entity-denoting nouns, can license arguments. In this section, we will successively examine the preservation of argument structure in both types of nouns.

Two questions can be addressed regarding the argument structure of deverbal nouns that denote entities. The first concerns the nominalization of verbal arguments, as it is commonly assumed that entity nominalizations refer to event participants denoted by an argument of their base verbs. For example, a verb that assigns the role of agent to its subject can derive an agent noun (e.g., *teach* → *teacher*), and a verb that assigns the role of result to its object can derive a result noun (e.g., *sculpt* → *sculpture*). However, argument nominalization is not always observed in our database, since 1721 (44%) of the non-figurative entity-denoting nouns have a relational type that does not correspond to any of the semantic roles assigned by the base verb to its arguments. Examples of these mismatches are presented in (18).

(18)
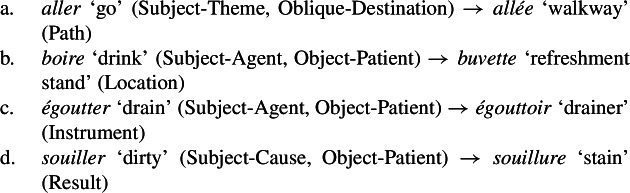

A particular case occurs when a verb with a cause subject derives an agent noun, as in *tuer* ‘kill’ → *tueur* ‘killer’ and *guérir* ‘heal’ → *guérisseur* ‘healer’. In this case, the semantic divergence between the base verb and the derived noun results from the specification of intentionality at the nominal level, in relation to the description of animate entities.

**Fig. 9:**
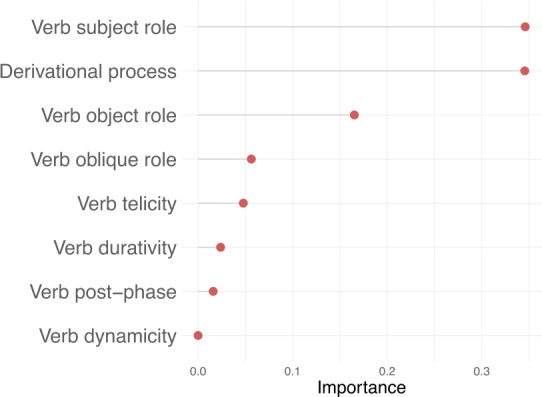
Variable importance for predicting argument nominalization in gradient boosting tree models

We conducted chi-squared tests to determine whether the ability to denote a participant that is not encoded as an argument of the base verb is significantly associated with morphological processes and the semantic properties of base verbs. All associations turned out to be statistically significant (*p* < .005), although effect sizes varied, with Cramér’s *V* ranging from .05 to .48 (*M* = .25, *SD* = .16). Further analysis using a gradient boosting tree classifier with 10-fold cross validation indicated strong predictive accuracy in aggregated results (.821). Predicting argument nominalization based on morphological processes and verbal properties significantly outperformed the No Information Rate baseline (.563, *p* < .001). Additionally, we analyzed the contribution of the different variables to the prediction, which revealed that the most influential predictors are the semantic role assigned to the verb subject and the morphological process involved (see Fig. 9). The ability of deverbal nouns to denote event participants that are not expressed by verbal arguments is clearly an effect of derivational patterns, and should be considered as such when describing nominalization processes.

The second question concerning argument preservation in entity nominalizations is whether the arguments of the nouns are inherited from the base verbs. In SONDE, there are 540 non-figurative entity-denoting nouns that license arguments. The argument structure of these nouns is always restricted to a single argument, which is systematically inherited from the base verbs. Specifically, the argument of the noun is assigned the same semantic role as the object or oblique argument of the verb. As previously mentioned, entity nominalizations with argument structure are semantically homogeneous, since Animate-agent largely prevails as their semantic type (89%), followed by Animate-beneficiary and Animate-pivot (3% each). We focused on the dominant semantic type and analyzed the 1919 Animate-agent nouns in the database to determine which factors condition their compatibility with arguments. Specifically, we examined how morphological processes and the semantic properties of base verbs relate to the presence of argument structure in Animate-agent nouns. Chi-squared tests revealed significant associations for all variables (*p* < .05) except verb dynamicity, with negligible to moderate effect size (Cramér’s *V* = [.06,.35], *M* = .18, *SD* = .11). In particular, important differences were observed across morphological processes. While suffixes such as -*euse* and -*rice* produce Animate-agent nouns that license an argument in half or more cases, others, such as -*ier*, -*ière*, -*ard*, and -*arde*, almost exclusively form agent nouns without argument structure.

A complementary analysis using a gradient boosting tree model yielded a prediction accuracy of .768 for argument preservation, only slightly improving the baseline of .750 (*p* = .04). Despite significant preferences, the association of agent nouns with arguments is poorly predicted based on verb properties and morphological processes, in the context of a widespread lack of argument structure. Additional factors might be considered to explain the argumental properties of Animate-agent nouns, such as the distinction between different types of agents (Benveniste, 1948; Rappaport Hovav & Levin, 1992; Roy & Soare, 2012; Huyghe & Wauquier, 2020; a.o.). For instance, it can be hypothesized that nouns denoting occasional agents (i.e., agents involved in a particular event) are more likely to license arguments than nouns denoting functional agents (i.e., agents defined by their occupational status).

The preservation of argument structure in eventuality nominalizations is more complex than in entity nominalizations. The argument structure of the base verb may not be fully preserved, not only in terms of the number of arguments but also with respect to their semantic roles. Among the 4152 non-figurative eventuality denoting nouns in SONDE, 3414 nouns (82%) preserve the argument structure of their base verb—including both the number of arguments and their semantic roles—whereas 738 nouns (18%) do not. Cases of non-preservation involve argument deletion or semantic role change, as illustrated for subjects in (19) and (20), respectively.

(19)

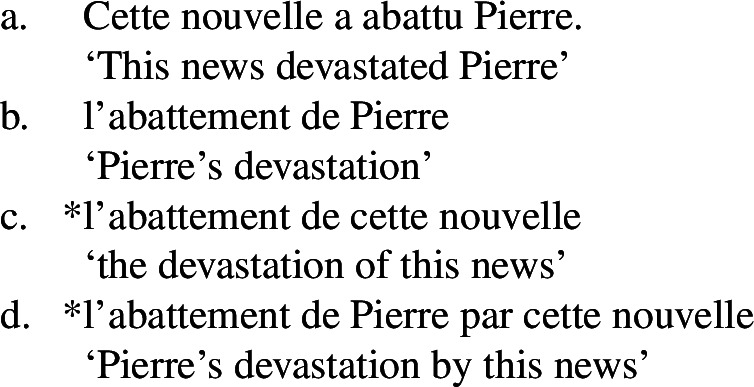

(20)
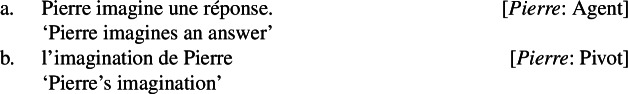

While preservation rates are consistent across argument types, the proportion of argument deletion and semantic role change varies by argument type. This variation is detailed in Table 16 for all specified arguments.

**Table 16:** Preservation of arguments from base verbs in eventuality nominalizations

	Subject	Object	Oblique
Preservation	3549 (85.5%)	2255 (85.7%)	536 (85.1%)
Argument deletion	364 (8.8%)	330 (12.5%)	87 (13.8%)
Semantic role change	239 (5.8%)	46 (1.7%)	7 (1.1%)

**Fig. 10:**
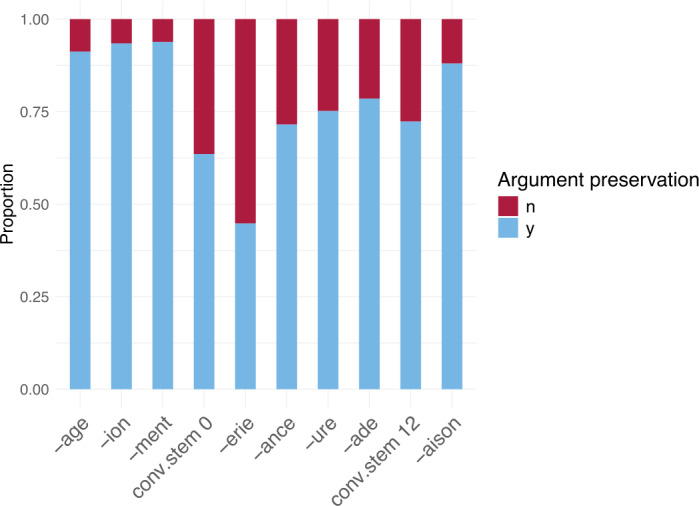
Argument preservation in the 10 most frequent processes forming eventuality nouns. Processes are ordered from left to right by decreasing frequency

One may ask whether the preservation of argument structure depends on morphological processes and the semantic properties of base verbs. According to chi-squared tests, these factors are significantly associated with argument preservation (*p* < .05), with Cramér’s *V* ranging from .03 to .43 (*M* = .15, *SD* = .12). The strongest association is found with morphological processes, following the tendencies illustrated in Fig. 10 for the 10 most represented processes. These tendencies closely align with those observed for aspectual preservation (see Fig. 7), and there is a strong correlation in the ability of processes to preserve lexical aspect and argument structure (Kendall’s *τ* = .78, *p* < .001). We trained a gradient boosting tree classifier to predict argument preservation based on morphological processes and verb properties. As in the case of aspectual preservation, the accuracy of the model (.845) was only slightly better than the high No Information Rate baseline (.822), although the improvement was statistically significant (*p* < .001).

**Fig. 11:**
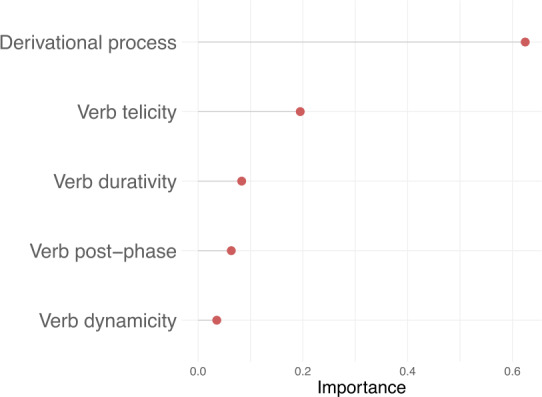
Variable importance for predicting argument shifts in gradient boosting tree models

To better understand the conditions under which eventuality nominalizations fail to preserve the argument structure of their base verbs, we analyzed the variation in argument shifts from verb to noun. The 10 most frequent types of shifts—identified through combinations of subject, object, oblique deletion and role change—are presented in Table 17, accounting for 90% of all cases of non-preservation. Chi-squared tests revealed significant associations (*p* < .01) between these argument shifts and both morphological processes and the aspectual features of base verbs, with a moderate effect size (Cramér’s *V* = [.19, .35], *M* = .25, *SD* = .07). A gradient boosting tree model predicting argument shifts based on these factors achieved a low accuracy of .353, still significantly outperforming the baseline of .210 (*p* < .001). The prediction was mainly driven by the distinction between the different morphological processes, as evidenced by predictor importance analysis (see Fig. 11). Overall, our findings suggest that the preservation of arguments between verbs and nouns is at least partially determined by derivational patterns. Beyond lexical idiosyncrasies, nominalization processes appear to be capable of modifying argument structure when deriving eventuality-denoting nouns.

**Table 17:** Most frequent argument shifts between base verbs and derived nouns. Verbs are characterized by their argument structure (AS), considering subject (Subj), object (Obj), and oblique (Obl) arguments. Argument shifts consist of either argument deletion (Del) or semantic role change (Cha)

Verb AS	Argument shift	Frequency	Example
Subj-Obj	Subj.Del-Obj.Del	140	*X* *finance Y* ‘X finances Y’
			→ la finance *de X/*de Y ‘the finance of X/Y’
Subj-Obj	Obj.Del	102	*X chuchote Y* ‘X whispers Y’
			→ le chuchotis de X/*de Y ‘the whispering of X/Y’
Subj	Subj.Cha	96	*X* (Theme) *bouge* ‘X moves’
			→ *la bougeotte de X* (Pivot) ‘the restlessness of X’
Subj	Subj.Del	82	*X jardine* ‘X gardens’
			→ le jardinage *de X ‘the gardening of X’
Subj-Obj	Subj.Cha-Obj.Del	68	*X* (Experiencer) *prévoit Y* ‘X foresees Y’
			→ *la prévoyance de X* (Pivot)/*de Y ‘the foresight of X/Y’
Subj-Obj	Subj.Del	59	*X affole Y* ‘X panics Y’
			→ l’affolement de Y/*de X ‘the panic of Y/X’
Subj-Obj	Subj.Cha	39	*X* (Cause) *gouverne Y* ‘X rules Y’
			→ *la gouvernance de X* (Agent)*/de Y* ‘the governance of X/Y’
Subj-Obj	Subj.Del-Obj.Cha	37	*X courbe Y* (Patient) ‘X bends Y’
			→ *la courbure de Y* (Pivot)/*de X ‘the curve of Y/X’
Subj-Obl	Subj.Cha-Obl.Del	24	*X* (Agent) *condescend à Y* ‘X condescends to Y’
			→ *la condescendance de X* (Pivot)/*à Y ‘the condescension of X/to Y’
Subj-Obl	Obl.Del	19	*X rêve de Y* ‘X dreams of Y’
			→ la rêverie de X/*de Y ‘the daydream of X/Y’

### Morphological families

As described in Sect. 2.1, verb-noun pairs in SONDE were sampled to form complete morphological families, ensuring that all nouns derived from each verb in the database were included. In this section, we examine the internal structure of these families, first by analyzing patterns of co-occurring semantic types within families (Sect. 3.4.1), and then by discussing cases in which identical types are assigned to multiple derivatives of the same verb (Sect. 3.4.2).

#### Semantic type co-occurrence

**Fig. 12:**
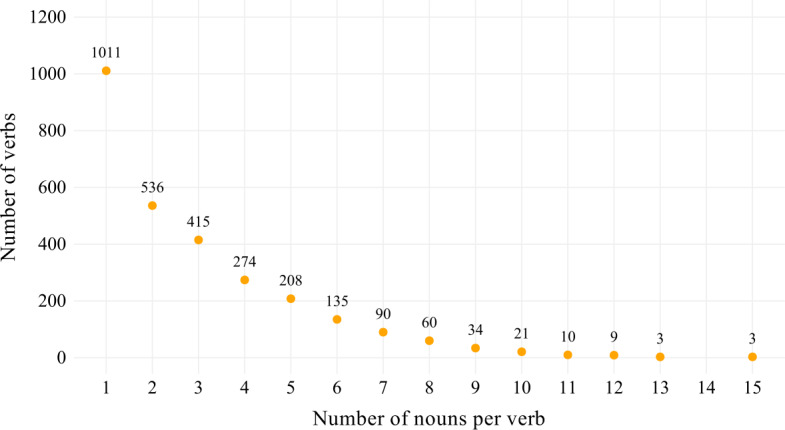
Distribution of verb lexemes by number of derived noun lexemes

Derived nouns are not evenly distributed across verbs in SONDE. The number of derivatives per verb, considering the 2809 verbal and 8202 nominal lexemes in the database, ranges from 1 to 15. As shown in Fig. 12, the number of base verbs decreases as the number of derivatives increases, with an average of 2.9 nominal lexemes per verbal lexeme (*SD* = 2.2). One may ask how derivatives are distributed across verbs based on their semantic type, and whether certain combinations of semantic types recur within deverbal families. Detecting such combinations is essential to uncover potential paradigmatic relationships within families of verb-related nouns.

The notion of derivational paradigm has been used in morphological studies to analyze the complex semantic relations and interdependence between members of a derivational family (van Marle, 1984; Bauer, 1997; Pounder, 2000; Štekauer, 2014; Bonami & Strnadová, 2019; Hathout & Namer, 2022; a.o.). Derivational paradigms can account for back-formation, cross-formation and multimotivation in complex words (Becker, 1993; Roché, 2011; Bonami & Guzmán Naranjo, 2023). They also shed light on the structural similarities between inflection and derivation (Stump, 1991; Blevins, 2001; Boyé & Schalchli, 2016), and support the idea that inflection and derivation exist along a continuum (Spencer, 2013; Štekauer, 2015; Copot et al., 2022). A key challenge in the paradigmatic approach is identifying sets of words that align in their semantic types across derivational families, at an appropriate level of semantic granularity. This task is further complicated by the existence of gaps in morphological families and the fact that derivational paradigms may exhibit a high degree of defectiveness (Antoniova & Štekauer, 2015; Stump, 2019; Sims, 2023).

To explore patterns of co-occurring semantic types in deverbal noun families and to identify consistent subfamilies based on their semantic structure, we adopted a probabilistic approach. Specifically, we used latent class analysis (LCA), which is a model-based clustering method that assumes the existence of unobserved (latent) categorical variables partitioning the observed data (Hagenaars & McCutcheon, 2002; Weller et al., 2020; Bauer, 2022). LCA estimates the probability that each observation belongs to a given latent class according to its response pattern, thereby revealing underlying hidden structures. By using LCA, we aimed to identify homogeneous subgroups of verbs based on the meaning of their derived nouns and to uncover latent paradigmatic structures within derivational families. The analysis cannot only distinguish latent classes among deverbal families, but also characterize each class by a probability distribution of nominal semantic types, thus allowing for an estimation of their potential defectiveness.

To apply LCA to our data, we extracted all unique pairings of verbal lexemes and nominal semantic types from SONDE, excluding semantic types representing less than 0.5% of the data. This yielded a dataset of 2696 verbal lexemes associated with 22 distinct nominal types (covering 8018 nominal lexemes). The data were then transformed into a binary matrix indicating the presence or absence of each nominal semantic type for each verbal lexeme. We fitted LCA models with a range of fixed latent class numbers, starting from 1 and increasing until models failed to converge, while using 20 replications for each model (as recommended by Sinha et al., 2021). The optimal model, determined by the lowest Bayesian Information Criterion (BIC), was the 5-class model. We assessed the quality of the classification by calculating its relative entropy, which was .85, indicating a clear separation between the latent classes. The accuracy of the classification was also evaluated by calculating the average posterior class probabilities, which were obtained by averaging the model-estimated class membership probabilities for the verbs most likely assigned to each class. These probabilities were high, ranging from .82 to .98 across the 5 classes (*M* = .89). Table 18 shows the number of distinct verbs in each class, determined by their dominant membership probabilities.

**Table 18:** Distribution of verbs across classes in latent class analysis, based on dominant membership probability

	Class 1	Class 2	Class 3	Class 4	Class 5
Number of verbs	348 (13%)	199 (7%)	1127 (42%)	630 (23%)	392 (15%)

**Fig. 13:**
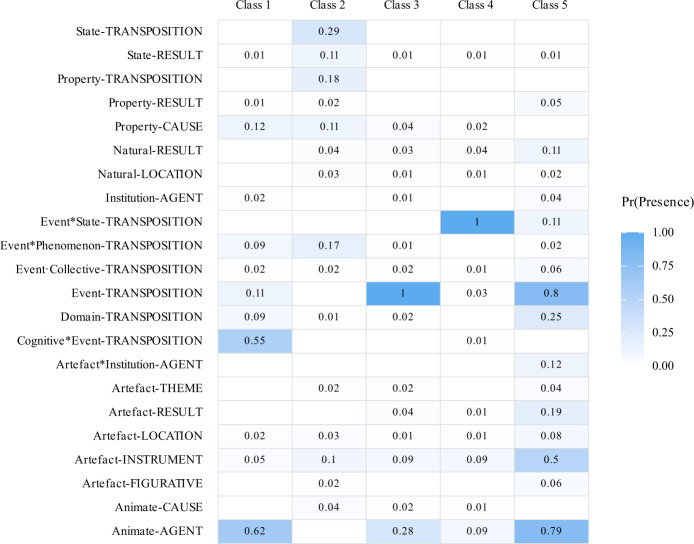
Estimated class-conditional probabilities of nominal semantic types in latent class analysis

The conditional probabilities of the nominal semantic types found in each class are presented in Fig. 13. Most semantic types have little to no probability of occurring in specific classes, making relatively low probabilities still characteristic of certain classes. Class 1 includes verbs with more than 50% chance of having in their derivational family nouns that denote an Animate-agent or a Cognitive*Event-transposition. Class 2 is characterized by the lowest representation of distinctive semantic types, with the highest probability corresponding to State-transposition at 29%, followed by Property-transposition at 18%, and Event*Phenomenon-transposition at 17%. Classes 3 and 4 are centered around a single dominant semantic type that is necessarily instantiated—Event-transposition in Class 3 and Event*State-transposition in Class 4—with Animate-agent in Class 3 being the only other semantic type that co-occurs with notable frequency. Finally, verbs in Class 5 have the most diverse nominal families, with high probabilities of being associated with nouns typed as Event-transposition, Animate-agent, and to a lesser extent Artefact-instrument, Domain-transposition and Artefact-result. The latent class analysis thus reveals that 5 subgroups can be distinguished among deverbal families depending on their semantic organization. Aligned semantic paradigms within each subfamily are structured by a few distinctive semantic types, some of which may be highly defective even within specific subgroups.

**Fig. 14:**
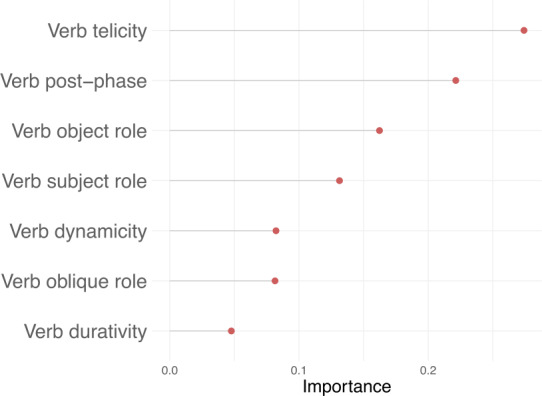
Variable importance for predicting latent derivational classes in gradient boosting tree models

To further analyze the properties of the deverbal subfamilies identified by LCA, we examined the semantic properties of the verbs in each group, considering their dominant probability of class membership (as indicated in Table 18). Chi-squared tests indicated significant associations between the classification of the verbs and all their aspectual and role-assignment properties (*p* < .001), with effect sizes ranging from moderate to strong (Cramér’s *V* = [.19,.70], *M* = .41, *SD* = .21). Together, these properties provide a reasonable prediction of class membership. A gradient boosting tree model predicting latent derivational classes based on the verbs’ semantic properties achieved an accuracy of .663, significantly outperforming the baseline of .418 (*p* < .001). An additional analysis of predictor importance revealed that the prediction is primarily driven by telicity and post-phase, followed by the semantic roles assigned to subjects and objects, as illustrated in Fig. 14.

More specifically, verbs in Classes 1 and 2 are predominantly atelic (53% and 74%, respectively), whereas verbs in Classes 3 and 5 are largely telic (65% and 64%, respectively), and most verbs in Class 4 exhibit variable telicity (65%). Class 4 also stands out in terms of post-phase properties, with 68% of its verbs marked by post-phase (vs. less than 8% in all other classes). This combination of variable telicity and post-phase in Class 4 aligns with the strong presence of Event*State-transposition nouns, in derivational families where other semantic types are poorly represented. Verb telicity in Classes 3 and 5 correlates with the prevalence of Event-transposition nouns within derivational families, whereas atelicity can be related to the formation of nouns that denote events with a cognitive facet in Class 1 and stative eventualities in Class 2. The most notable differences between verbs in Class 1 and Class 2 lie in the roles assigned to subjects, since agent subjects dominate in Class 1 (89%), whereas a higher proportion of pivot (28%), cause (23%), and experiencer (12%) subjects is observed in Class 2. These differences are consistent with the distinctive presence of State-transposition and Property-transposition nouns among derivatives in Class 2, which contrasts with the important probabilities of Animate-agent and Cognitive*Event-transposition nouns in Class 1. As for Class 3 and Class 5, they differ in that Class 3 includes more intransitive verbs (32% vs. 14%) and fewer verbs with patient objects (21% vs. 46%). This contrast may explain why verbs in Class 5 show a greater tendency to form agent-, instrument-, and result-denoting nouns than verbs in Class 5. Overall, these findings suggest that the semantic properties of verbs influence the semantic structure of their derivational families. Accordingly, groups of verbs can be distinguished based on the correlation between their aspectual and argumental properties and the patterns of meaning found in their derived nouns.

#### *N*-tuples

Certain semantic types within morphological families are assigned to more than one derivative, which typically occurs when different morphological processes apply to the same base to produce (nearly) synonymous words. SONDE contains 1007 *n*-tuples, defined as sets of two or more nouns that share the same combined semantic type and are derived from the same verb meaning. These *n*-tuples encompass 2122 nominal lexemes (26% of all nominal lexemes in the database) and are related to 780 distinct verbal lexemes. They consist of 907 doublets, 92 triplets, 8 quadruplets, and instantiate 55 distinct semantic types. However, the distribution is uneven, as more than half of these types have only one *n*-tuple. The 10 most frequent semantic types, accounting for 92% of all *n*-tuples, are presented in Table 19. Similarly, 193 different combinations of morphological processes are identified among the *n*-tuples, also showing a skewed distribution. The 10 most common processes, observed in 52% of the cases, are listed in Table 20.

**Table 19:** Most frequent semantic types instantiated by *n*-tuples in SONDE

Semantic type	# *n*-tuples	Examples
Event-transposition	362	*gesticulage*, *gesticulation* ‘gesticulation’
Animate-agent	231	*débatteuse*, *débattrice* ‘debater’
Event*State-transposition	123	*déclin*, *déclinement* ‘weakening’
Artefact-instrument	95	*calculatrice*, *calculette* ‘calculator’
Cognitive*Event-transposition	35	*fanfaronnade*, *fanfaronnerie* ‘boast’
Event*Phenomenon-transposition	30	*gazouillement*, *gazouillis* ‘chirping’
Artefact-result	15	*damasserie*, *damassure* ‘pattern-welded steel’
Natural-result	13	*crachat*, *crachis* ‘spit’
Domain-transposition	10	*flibuste*, *flibusterie* ‘buccaneering’
Property-cause	9	*gouaille*, *gouaillerie* ‘cockiness’

**Table 20:** Most frequent process rivalries instantiated by *n*-tuples in SONDE

Competing processes	# *n*-tuples	Examples
-*age*, -*ment*	313	*bétonnage*, *bétonnement* ‘concreting’
-*eur*, -*ier*	28	*placeur*, *placier* ‘usher’
-*eure*, -*euse*	28	*manageure*, *manageuse* ‘manager’
-*eur*, -*oir*	26	*aiguiseur*, *aiguisoir* ‘sharpener’
-*ant*, -*eur*	25	*exécutant*, *exécuteur* ‘executor’
-*ment*, -*is*	24	*crachotement*, *crachotis* ‘spluttering’
-*age*, -*is*	20	*gribouillage*, *gribouillis* ‘scribble’
-*age*, -*ment*, -*is*	20	*gratouillage*, *gratouillement*, *gratouillis* ‘scratching’
-*ard*, -*eur*	20	*pleurnichard*, *pleurnicheur* ‘whiner’
-*age*, conversion 0	16	*labourage*, *labour* ‘ploughing’

Nouns derived from the same base verb and sharing the same semantic type may differ in their lexical aspect and argument structure. A detailed examination of the *n*-tuples in SONDE reveals that 96 of them exhibit such differences. For example, *poussée* ‘thrust’ is an activity nominalization whereas *poussette* ‘push’ is an achievement nominalization, and *chatouillage* ‘tickling’ differs from *chatouillis* ‘tickle’ by licensing a beneficiary argument in addition to the agentive argument. However, the vast majority of *n*-tuples remain consistent in their lexical aspect and argument structure, as 911 (90%) show no differences according to the semantic features described in the database. Yet this does not imply that semantic distinctions are necessarily absent from those *n*-tuples. Specialization may occur at the lexical level, beyond derivational semantics, adding features that refine compositional meanings and create semantic differentiation. Such idiosyncratic lexical features can be found in Artefact-instrument and Animate-agent doublets with distinct onomasiological functions. For example, while *sarcleur* and *sarcloir* both refer to weeding tools, *sarcloir* typically denotes a manual instrument, whereas *sarcleur* is generally used for a larger mechanical device operated with a vehicle. Similarly, *gouvernant* and *gouverneur* both denote individuals who hold positions of power, but *gouvernant* ‘ruler’ broadly designates a person who exercises political authority, whereas *gouverneur* ‘governor’ is more specific, referring to the head of a financial institution or the leader of a state in certain countries.

Apart from semantic differentiation, factors such as textual genre, style, register, and sociolinguistic influences may contribute to the distinction between *n*-tuples, as they can play a role in resolving morphological competition (see, e.g., Guz, 2009; Säily, 2011; Naccarato, 2019). For instance, *comprenette* is certainly more informal than *compréhension* when denoting the faculty of understanding, and *logis* may be more literary than *logement* when referring to a dwelling. Nevertheless, many *n*-tuples remain difficult to differentiate, especially in some of the most frequent instances of affix rivalry, such as the competition between -*age* and -*ment*. No clear difference was found between doublets like *ajustage* and *ajustement* (‘adjusting’), *butinage* and *butinement* (‘foraging’), *déneigeage* and *déneigement* (‘snow clearing’), *empalage* and *empalement* (‘impalement’), *épluchage* and *épluchement* (‘peeling’), etc.—confirming earlier findings by Fradin (2016) on the equivalence of certain doublets in -*age* and -*ment* in French. The existence of such indistinguishable doublets parallels a similar phenomenon in inflection, where enduring cases of overabundance occur without any differentiation in use or historical evolution (Fehringer, 2004; Thornton, 2012; Cappellaro, 2013). Further research is still required to determine the exact proportion of true synonyms among deverbal *n*-tuples and to assess the importance of derivational overabundance.

## Conclusion

In this article, we introduced SONDE, a large-scale semantically annotated database of French deverbal nouns. We first outlined the methodology used to create the database, including the sampling method, the semantic categories for describing deverbal nouns, and the annotation process. We then exploited the database to address key research questions about the semantics of verb-to-noun derivation, focusing on the form-meaning mapping in nominalizations, the transfer of cross-categorial semantic properties between verbs and nouns, and the organization of deverbal nouns in morphological families.

The exploration of the database yielded a wide range of descriptive results, shedding light on the semantic diversity and ambiguity of deverbal nouns, the distribution and correlation between their multiple semantic properties, the polyfunctionality and similarity of deverbal processes, the aspectual and argumental properties of bases and derivatives, and the distribution of semantic types across deverbal families. Further data analysis provided important theoretical insights into the semantic aspects of verb-to-noun derivation. First, it was found that a network of semantic functions underlies the diversity of deverbal processes. This network is partially shaped by semantically motivated relationships and is organized around a few core semantic functions. The relationships between functions influence semantic similarity between processes and define consistent groups of processes. Accordingly, the one-to-many and many-to-one mappings between form and meaning in verb-to-noun derivation are not independent, symmetrical relationships. Semantic polyfunctionality affects semantic similarity in two ways. On the one hand, the degree of similarity between processes depends on the proportion and realization frequency of shared functions. On the other hand, the mechanisms of semantic extension that drive polyfunctionality contribute to the emergence of similarities between processes.

Another finding was that the aspectual and argumental properties of base verbs are not necessarily preserved through nominalization, even in nouns that denote eventualities. Furthermore, semantic discrepancies between base verbs and derived nouns cannot simply be attributed to idiosyncratic lexicalization effects. The non-preservation of base properties is influenced by derivational patterns, making it an inherent part of morphological processes. Derivation can contribute to the elaboration of aspectual properties within the nominal domain, which is congruent with the existence of strictly nominal aspectual categories and eventuality types, such as Domain and Property.

The analysis of deverbal families revealed notable variations in the availability of nominal semantic types depending on the base verb. Distinct family structures can be identified based on the alignement of semantic types across families, with varying degrees of defectiveness. These structures are largely determined by the semantic properties of the base verbs. Deverbal families also include a considerable number of *n*-tuples, whose potential indistinguishability may not be accidental, but rather an inherent consequence of morphological productivity.

Altogether, these results show that the data collected in SONDE provide a comprehensive basis for exploring the semantic aspects of verb-to-noun derivation. Large-scale morphological resources, enriched with detailed semantic descriptions, are essential for addressing fundamental research questions regarding the semantics of derivation. The abundance of data and broad empirical coverage allow for the application of adapted metrics, statistical testing, and quantitative generalizations. They also support a probabilistic approach to many issues related to the semantics of derivational processes, which is arguably the most accurate method for studying these linguistic phenomena. To achieve the level of semantic precision needed for an in-depth analysis of deverbal nouns and processes, manual annotation was necessary, as automatic methods currently lack the ability to provide the required information. In particular, distinguishing and pairing the various meanings of bases and derivatives was crucial for a comprehensive analysis of the semantic aspects of verb-to-noun derivation. While the subjectivity and potential unreliability of manual annotation can be controlled through careful procedures and evaluation of inter-annotator agreement, evident limitations remain. Manual annotation is highly time consuming and requires advanced linguistic expertise. It is also typically restricted to one or a few languages, which inevitably limits the scope of the findings. In practice, applying detailed manual annotation methods like those developed in SONDE to a wide range of languages is unrealistic. Nevertheless, thorough language-specific descriptions may reveal underlying structures and organizational principles, informing advanced research hypotheses that can subsequently be tested cross-linguistically.

In this paper, we performed only a subset of the analyses that can be conducted using SONDE. Many other applications are possible, including qualitative analyses based on the examples provided for each verbal and nominal meaning in the database, or focused investigations into specific semantic roles, reflexive verb forms, collective nouns, feminine agent nouns, opaque sense extensions, among others. Additionally, data from SONDE can be combined with computational methods to explore specific issues (e.g., semantic granularity in derivation), or serve as a gold-standard for training classifiers or machine annotators. By making the resource publicly available, we hope that other researchers will use it for their own studies and benefit from the effort invested in its development.

## Data Availability

All supplementary materials for this paper, including the SONDE database, annotation guidelines, analysis scripts, and additional figures, are publicly available on the Open Science Framework platform at: https://osf.io/feuz7.

## Footnotes

1 SONDE is the acronym for Semantics Of Nouns DErived from verbs.

2 To address lemmatization issues and the potential amalgamation of masculine and feminine nouns under lemmatized forms, all the selected nouns ending in -*ant*, -*ard*, -*eur*, -*ier* and -*on* were manually reviewed for feminine counterparts ending in -*ante*, -*arde*, -*euse*, -*eure*, -*rice*, -*ière* and -*onne* in the reference corpus. When such forms were found, they were included in the database as separate entries. Similarly, nouns ending in -*aire* were examined for both masculine and feminine forms, and double entries were created when both forms were attested as nouns in the corpus.

3 For the remainder of the article, the following typographical conventions are used: ontological types are capitalized (e.g., Event, Artefact), while relational types are presented in small caps (e.g., instrument, result).

4 An inherent limitation of the database is the lack of information on sense frequency, which would make it possible to distinguish between rare and dominant senses and to weight observations regarding the distribution of semantic types across derivatives. Collecting such data, for instance through detailed annotation of random corpus samples for each noun and verb, would have required considerable additional effort and was beyond the scope of the project.

5 Confusion matrices showing annotation agreements and disagreements are provided in the supplementary materials.

6 Fisher’s exact test was preferred to the chi-square or a G test because of very low expected values for various pairs of features (see McDonald, 2014).

7 An odds ratio greater than 1 indicates that the two functions co-occur more frequently than expected under independence, meaning that the presence of one function increases the probability of the other.

8 Percentage similarity is calculated from Equation (1), where *T* is the set of semantic types, and $N_{p}^{t}$ is the number of derivatives of type *t* formed with process *p*.

1 $$ PS = \frac{2 \sum _{t \in T} \min \left (N_{p_{1}}^{t}, N_{p_{2}}^{t}\right )}{\sum _{t \in T} \left (N_{p_{1}}^{t} + N_{p_{2}}^{t}\right )} $$

9 Motivated similarity is calculated from Equation (2), where *F(p)* is the set of semantic functions of process *p*, and *E(p)* is the subset of *p*’s functions that result from the semantic extension of *p*’s functions.

2 $$ MS = \frac{| E(p_{1}) \cap F(p_{2}) | + | E(p_{2}) \cap F(p_{1}) |}{2 \, | F(p_{1}) \cap F(p_{2}) |} $$

10 The mass/count distinction is associated with a form of boundedness that does not have an equivalent in the verbal domain (Huyghe, 2011). Contrary to common assumptions, the mass/count distinction does not correspond to telicity, since count nouns can be telic or atelic depending on whether they denote an event that includes a culmination or not (e.g., *accouchement* ‘delivery’ vs. *manifestation* ‘demonstration’). The mass/count distinction does not align either with perfective vs. imperfective grammatical aspect or bounded vs. unbounded actualization aspect (Declerck, 2007), as count nouns are compatible with event representations that may or may not include temporal boundaries (e.g., *une discussion en cours* ‘an ongoing discussion’ vs. *la discussion d’hier* ‘yesterday’s discussion’). The mass/count distinction relates to the description of spatio-temporally individuated actions, which in the verbal domain is realized at the sentential level, depending upon the denotation of occurrences of actions (e.g., *Elle a jardiné ce matin* ‘She gardened this morning’) as opposed to habits and action types (e.g., *Elle jardine le matin* ‘She gardens in the morning’, *Elle adore jardiner* ‘She loves gardening’).
